# An intellectual-disability-associated mutation of the transcriptional regulator *NACC1* impairs glutamatergic neurotransmission

**DOI:** 10.3389/fnmol.2023.1115880

**Published:** 2023-07-14

**Authors:** James A. Daniel, Sofia Elizarova, Ali H. Shaib, Abed A. Chouaib, Helge M. Magnussen, Jianlong Wang, Nils Brose, JeongSeop Rhee, Marilyn Tirard

**Affiliations:** ^1^Department of Molecular Neurobiology, Max Planck Institute for Multidisciplinary Sciences, Göttingen, Germany; ^2^Institute for Neuro- and Sensory Physiology, University Medical Center Göttingen, Göttingen, Germany; ^3^Department of Cellular Neurophysiology, Center for Integrative Physiology and Molecular Medicine (CIPMM), Saarland University, Homburg, Germany; ^4^MRC Protein Phosphorylation and Ubiquitination Unit, School of Life Sciences, University of Dundee, Dundee, United Kingdom; ^5^Department of Medicine, Columbia Center for Human Development and Stem Cell Therapies, Columbia University Irving Medical Center, New York, NY, United States

**Keywords:** SUMO, neuron, NAC1/BTBD14B, synapse, SynGAP1

## Abstract

Advances in genome sequencing technologies have favored the identification of rare *de novo* mutations linked to neurological disorders in humans. Recently, a *de novo* autosomal dominant mutation in *NACC1* was identified (NM_052876.3: c.892C > T, NP_443108.1; p.Arg298Trp), associated with severe neurological symptoms including intellectual disability, microcephaly, and epilepsy. As *NACC1* had never before been associated with neurological diseases, we investigated how this mutation might lead to altered brain function. We examined neurotransmission in autaptic glutamatergic mouse neurons expressing the murine homolog of the human mutant NACC1, i.e., Nacc1-R284W. We observed that expression of Nacc1-R284W impaired glutamatergic neurotransmission in a cell-autonomous manner, likely through a dominant negative mechanism. Furthermore, by screening for Nacc1 interaction targets in the brain, we identified SynGAP1, GluK2A, and several SUMO E3 ligases as novel Nacc1 interaction partners. At a biochemical level, Nacc1-R284W exhibited reduced binding to SynGAP1 and GluK2A, and also showed greatly increased SUMOylation. Ablating the SUMOylation of Nacc1-R284W partially restored its interaction with SynGAP1 but did not restore binding to GluK2A. Overall, these data indicate a role for Nacc1 in regulating glutamatergic neurotransmission, which is substantially impaired by the expression of a disease-associated Nacc1 mutant. This study provides the first functional insights into potential deficits in neuronal function in patients expressing the *de novo* mutant NACC1 protein.

## Introduction

Nucleus accumbens protein 1 (NACC1 in human; Nacc1 in rodent) is a ubiquitously expressed but functionally enigmatic protein that has been linked to multiple cellular processes, most distinctly to cell cycle control and tumorigenesis. It contains a BTB/POZ domain and is primarily localized to cell nuclei (Korutla et al., 2005; Shen et al., 2007; Tatemichi et al., 2015), where it acts as a transcriptional regulator (Korutla et al., 2002, 2005, 2009; Nakayama et al., 2007; Okazaki et al., 2012; Gao et al., 2014, 2020). In highly proliferating cells, NACC1 is subject to post-translational modification by SUMOylation (Schou et al., 2014; Hendriks et al., 2015, 2018). This process regulates NACC1 recruitment to nuclear promyelocytic leukemia protein (PML) bodies, but the functional consequences are unknown (Tatemichi et al., 2015).

Nacc1 was initially discovered as an immediate-early gene product that is upregulated in response to chronic cocaine exposure in the nucleus accumbens of rodent brains (Cha et al., 1997; Korutla et al., 2002; Wang et al., 2003). In neurons, Nacc1 is thought to support the activity-driven translocation of proteasome components from the cell nucleus to the dendritic spines of synapses (Shen et al., 2007). This led to the yet untested proposition that Nacc1 regulates synapse function in neurons. In accord with this, a heterozygous *de novo* mutation in the gene encoding NACC1 (*NACC1*, NM_052876.3: c.892C > T, NP_443108.1; p.Arg298Trp, referred here as NACC1-R298W for simplification purpose) was recently identified by whole-exome sequencing in eight unrelated individuals with a neurodevelopmental disorder characterized by severe intellectual and developmental disability, epilepsy, and abnormal brain morphology (Schoch et al., 2017; Lyu et al., 2021). These observations show that the pathogenic NACC1 mutant protein perturbs brain development and neural function. Interestingly, genetic deletion of Nacc1 in mice causes only a mild defect in vertebral development but no gross neurological or developmental consequences (Yap et al., 2013), indicating that NACC1-R298W may be a dominant-negative or gain-of-function variant.

*De novo* genetic mutations are a major cause of intellectual disability (de Ligt et al., 2012; Hamdan et al., 2014), often affecting genes associated with glutamatergic neurotransmission (Hamdan et al., 2011; Parker et al., 2015). In view of this general link and the neurological symptoms of patients expressing the NACC1-R298W variant, we explored whether the expression of NACC1-R298W in human patients might cause neurodevelopmental defects by perturbing neurotransmission. To this end, we determined the functional consequences and impact of Nacc1-R284W expression (the murine equivalent of NACC1-R298W) on excitatory synaptic transmission in mouse neurons. We report that Nacc1-R284W impairs glutamatergic neurotransmission in a dominant negative manner. We further discover new interaction partners of Nacc1, including the post-synaptic proteins SynGAP1 and GluK2A, and show that the R284W mutation inhibits Nacc1 binding to these proteins. Finally, we show that the R284W mutation causes striking hyper-SUMOylation of Nacc1 in neurons. These data provide the first insights into potential molecular and cellular defects underlying disease in human patients expressing NACC1-R298W.

## Materials and methods

### Animals

WT primary hippocampal neurons were prepared from C57BL/6N P0 neonatal mice. Nacc1-KO mice were generously provided by Jianlong Wang (Columbia University, New York City, USA). Nacc1-KOs were assessed and classified as unburdened according to standard ethical guidelines. Mouse breeding was performed with permission of the Niedersächsisches Landesamt für Verbraucherschutz und Lebensmittelsicherheit (LAVES). Animals were kept in groups according to European Union Directive 63/2010/EU and ETS 123. Mice were housed in individually ventilated cages (type II 8 superlong, 435 cm^2^ floor area; TECHNIPLAST), in specific pathogen-free conditions, at 21 ± 1°C, 55% relative humidity, under a 12 h/12 h light/dark cycle. Mice received food and tap water *ad libitum* and were provided with bedding and nesting material. Cages were changed once a week. Animal health was controlled daily by caretakers and by a veterinarian. Health monitoring (serological analyses; microbiological, parasitological, and pathological examinations) was done quarterly according to FELASA recommendations with either NMRI sentinel mice or animals from the colony. The mouse colony used for experiments did not show signs of pathogens. The sex of neonatal mice used for generating cultures was not checked.

### Mouse genotyping

For mouse genotyping, genomic DNA was extracted from tail biopsies using the Nexttech Genomic DNA isolation kit (Cat. No. 10.924). Primers for genotyping were 5’-CAGGGGCTGACA GTCATCTT-3′ (antisense primer, detects both Nacc1 WT and KO alleles), 5’-TGAGAAGGTAGAGGCCCTTCC-3′ (sense, detects only the Nacc1 WT allele), and 5’-CTGGGGAATGGATGGTTTTAA ATTTGG-3′ (sense, detects only the Nacc1-KO allele). These primers generate a 483 bp WT product and a 439 bp KO product. PCR reactions (20 μL) were performed using 1 U of MyTaq HS DNA polymerase (Bioline, BIO-21113) in MyTaq reaction buffer, 1 pmol/μL of each primer, and 0.25 mM of each dNTP, supplemented with 2.5 mM MgCl_2_. PCR reactions were performed using the following cycling conditions: 96°C for 3 min, 33 cycles of 94°C for 30 s, 62°C for 1 min, 72°C for 1 min, then finally 72°C for 7 min.

### DNA vectors

The long form of murine Nacc1 (NM_025788) in the plasmid pCMV6-Kan/Neo was purchased (Origene, Rockville, USA) and used as a template for all Nacc1 constructs. Nacc1 point mutations were generated via site-directed mutagenesis using a QuickChange Lightning kit and standard protocols (Agilent, Santa Clara, USA). N-terminal HA tags were added to Nacc1 constructs to facilitate biochemical identification. HA-Nacc1-WT, HA-Nacc1-K167R, HA-Nacc1-K485R, HA-Nacc1-K167R/K485R (HA-Nacc1-2KR), HA-Nacc1-R284W, and HA-Nacc1-R284W-2KR were expressed in the pcDNA3 vector. pEGFP-N1 was obtained commercially (Takara Bio, Kusatsu, Japan). Expression vectors encoding lentiviral supplementary proteins (pCMVdeltaR8.2 and VSV-G) and the pFUGW expression vector were described previously (Lois et al., 2002; Hsia et al., 2014). The pF(syn)-ugw-rbn vector, into which HA-Nacc1-WT and HA-Nacc1-R284W were cloned, was kindly provided by Christian Rosenmund (Charité, Berlin, Germany) (Trimbuch et al., 2014). This construct drives neuron-specific expression of the cDNA of interest under the control of the synapsin promoter, as well as the expression of EGFP under the control of the ubiquitin promoter (Hsia et al., 2014). The FUGW lentiviral vector lacks the synapsin promoter and served as a control ‘empty’ vector, with lentiviral infection simply resulting in the expression of EGFP driven by the ubiquitin promoter. For yeast two-hybrid screening, full-length mouse Nacc1 (long isoform) was expressed in pLexN as the bait construct, and the prey consisted of a rat cDNA library expressed in pVP16-3, which was a generous gift from Thomas Südhof (Stanford University, Stanford, USA). pcDNA3.1-GluK2A (Q edited form) was kindly provided by Christophe Mulle (University of Bordeaux, Bordeaux, France). cDNA encoding HA-SUMO1 and HA-SUMO2 were expressed in pCRUZ-HA and were kindly provided by Frauke Melchior (University of Heidelberg, Heidelberg, Germany). PRK5-SynGAP1α (myc-tagged) was a kind gift from Richard Huganir (Johns Hopkins University, Baltimore, USA).

### Yeast-two-hybrid screening

The LexA Yeast-Two-Hybrid system was used to screen a randomly primed cDNA brain library from postnatal day 8 rats for interactors of Nacc1, as described previously (Betz et al., 1997). Full-length mouse Nacc1 (long isoform) was amplified by PCR from pcDNA3-Nacc1 and subcloned into the EcoRI/BamHI sites of the yeast expression vector pLexN. The expression of this bait construct generates Nacc1-protein that is N-terminally linked to the DNA-binding domain of LexA and a SV40 large T-antigen nuclear localization signal. The cDNA rat brain library (‘prey’) was described previously (Betz et al., 1997). Bait and prey vectors were sequentially transformed into the L40 strain of *Saccharomyces cerevisiae* using lithium acetate (Betz et al., 1997). Screening YTH screens were performed as described (Vojtek et al., 1993). Activation of the HIS3 and LacZ selection genes though the bait construct with the empty prey plasmid alone was excluded in control experiments. In addition, 6 mM 3-Aminotriazole (3-AT), an inhibitor of the HIS3-gene product, was included in all yeast media for prevention of non-specific transactivation of the HIS3 gene during the screen (Jones and Fink, 1982).

Our screen covered the entire library, which was estimated to contain 5 × 10^6^ clones. Over three consecutive days, 400 positive clones were isolated and tested for ß-Galactosidase (ß-Gal) activity by filter assays (Vojtek et al., 1993). ß-Galactosidase is encoded by LacZ, the second reporter gene under the control of the LexA operator in the L40 yeast strain. Growth on histidine-omitted minimal media and the ß-Gal assay provide two independent reports on transcriptional activity of LexA-VP16 and, consequently, bait-prey interaction. To minimize false positives, only double-positive (His+/ß-Gal+) colonies were further processed. 150 double-positive yeast clones were cultured and the bait-plasmids eliminated by removal of the selective pressure on the *TRP1* gene, which facilitates the synthesis of tryptophan. The remaining DNA was obtained by phenol/chloroform/isoamylalcohol extraction and transformed into *E. coli*. pVP16-3-Prey plasmids contain an ampicillin resistance gene, whereas pLexN-bait plasmids contain a kanamycin resistance gene. Consequently, culturing transformed bacteria in ampicillin supplemented media eliminates residual bait-plasmids if present. pVP16-3 prey plasmids were prepared from individual bacterial clones and sequenced. The Basic Local Alignment Search Tool (BLAST) was used to identify the corresponding genes in the GenBank database (NCBI). All independent cDNA prey clones were then transformed again into the L40 yeast strain together with the pLexN-Nacc1 bait, or pLex-Lamin or empty pLexN as negative controls, and re-tested for ß-Gal activity to validate the specificity of the bait-prey interaction. Validated interactions were further confirmed by co-immunoprecipitation experiments. Initially, the SUMOylation deficient Nacc1, Nacc1-2KR, was also examined in the screen, but it exhibited very high activation of the reporter gene *HIS3* in the absence of prey plasmid. Due to this high level of auto-activation, we opted to focus on Nacc1-WT alone in the screen.

The screen yielded approximately 2000 *HIS3*-positive clones, of which 383 clones exhibited β-Galactosidase activity. 150 clones were selected for sequencing, of which 129 contained in-frame inserts comprised of 56 distinct sequences. The 56 prey clones were then amplified in *E. coli* under antibiotic selection, and re-transformed into yeast (along with the Nacc1-WT prey plasmid) and examined for *HIS3* and *LacZ* expression, resulting in a ‘validated’ list of 26 clones, encoding 16 proteins.

### Primary neuronal culture

Primary neurons were cultured from P0 mouse hippocampus, either as mass cultures or micro island cultures, as previously described (Burgalossi et al., 2012). Briefly, hippocampi of P0 pups were dissected and digested for 45 min at 37°C in 2.5 U/mL papain (Worthington Biomedical Corporation), 0.2 mg/mL L-cysteine (Sigma), 1 mM CaCl_2_, and 0.5 mM EDTA (in DMEM). After digestion, hippocampi were incubated for 15 min at 37°C in DMEM containing 10% heat-inactivated FBS, 2.5 mg/mL albumin, and 2.5 mg/mL trypsin inhibitor. The tissue was then triturated using a 200 μL pipette tip. Cells were either seeded on 35 mm round coverslips containing glial cells cultured as micro-islands (5,000 neuronal cells plated per well), or on 60 mm culture dishes coated with poly-L-lysine (one pair of mouse hippocampi per dish). Neurons were maintained at 37°C and 5% CO2 in Neurobasal A medium (Gibco) containing 2% B-27 (Gibco), penicillin (100 U/mL) and streptomycin (100 μg/mL). For mass cultures, neurons were washed twice in warm medium to remove cellular debris at DIV 1 and maintained in 6 mL of fresh medium as above. Neurons were maintained at 37°C and 5% CO_2_. For experiments using Nacc1-KO mice, WT littermates were prepared in parallel as controls.

### Lentivirus production and neuronal transduction

HEK293FT cells were transfected using Lipofectamine 2000 (Thermo Fischer Scientific). Lentiviral expression constructs were co-transfected with packaging and envelope vectors, as previously described (Follenzi and Naldini, 2002a,b; Lois et al., 2002; Salmon and Trono, 2006). 6 h after the addition of DNA-Lipofectamine preparations, the medium was removed and replaced with DMEM containing 2% FBS, penicillin/streptomycin (100 U/mL, 100 μg/mL), and 10 mM sodium butyrate. The cell medium (20 mL) was harvested 40 h later. Viral particles were concentrated to a final volume of 500 μL by filtering the medium using Amicon Ultra-15 centrifugal filters (100 kDa cut-off; Millipore), followed by washing the particles twice in Neurobasal-A medium and twice in tris-buffered saline. Concentrated lentivirus was then divided into aliquots, snap-frozen in liquid N_2_ and stored at −80°C. Autaptic and mass cultures were infected with lentivirus at DIV 1. Neurons were then left for at least 9 days prior to electrophysiological analysis to allow lentivirus infection and transgene expression.

### Culturing and transfection of cell lines

HEK293FT and N2A cell lines were maintained at 37°C and 5% CO_2_ in DMEM containing 10% heat-inactivated fetal calf serum, penicillin (100 U/mL), and streptomycin (100 μg/mL). For lentivirus production, HEK293FT cells were maintained in Geneticin (500 μg/mL). For overexpression experiments, cells were transiently transfected using Lipofectamine 2000 (Thermo Fisher Scientific, Waltham, USA). 12 μg of plasmid DNA were used per 100 mm circular dish. Cells were harvested for protein extraction 40 h after transfection.

### Immunoprecipitation

Anti-HA immunoprecipitation from brain samples of His_6_-HA-SUMO1 mice was performed as described (Tirard et al., 2012; Tirard and Brose, 2016; Daniel et al., 2017; Ripamonti et al., 2020). Briefly, proteins from powdered frozen brains were solubilized with buffer containing 150 mM NaCl, 20 mM Tris (pH 7.4), 1% Triton X-100, 0.5% sodium deoxycholate, and 0.1% SDS, protease inhibitors (1 μg/mL aprotinin, 0.5 μg/mL leupeptin, and 17.4 μg/mL phenylmethylsulfonyl fluoride), and 20 mM NEM (freshly dissolved in DMSO before addition). Brain lysate was centrifuged for 1 h at 100,000 × *g* at 4°C. HA-tagged proteins were enriched from cleared lysates on an anti-HA column, and bound proteins were eluted via HA peptide competition. For immunoprecipitation of target proteins from transfected cells, cells were removed from the growth surface using trypsin or gentle pipetting to dislodge the cells. Cells were then pelleted by centrifugation, resuspended in PBS, and pelleted again. The cell pellet was then resuspended in fresh ice-cold immunoprecipitation buffer (150 mM NaCl, 50 mM Tris, 1% Triton X-100, 0.1% SDS, 0.2% sodium deoxycholate, 20 mM N-ethylmaleimide, 1 μg/mL aprotinin, 0.5 μg/mL leupeptin, and 17.4 μg/mL phenylmethylsulfonyl fluoride). Lysis buffer was at pH 8 for anti-HA immunoprecipitations where cells were co-transfected with HA-Nacc1 and GluK2A. For all other experiments, the pH of the lysis buffer was 7.4. Samples were then sonicated, incubated on ice for 30 min and ultracentrifuged at 135,000 × *g* for 1 h. For anti-HA immunoprecipitation, anti-HA-conjugated agarose beads (monoclonal anti-HA agarose affinity gel, clone HA-7, Sigma-Aldrich) were added, and the mixture was incubated for 4 h at 4°C. For anti-myc immunoprecipitation, anti-myc-conjugated agarose beads (polyclonal anti-myc agarose affinity gel, A7470-1ML, Sigma-Aldrich) were used following the same protocol as for anti-HA beads. For enrichment of SynGAP1, cell lysate after centrifugation was combined with 3 μL (1.5 μg) of anti-SynGAP1 antibody and incubated for 4 h at 4°C. A 1:1 mixture of protein-G- and protein-A-conjugated sepharose beads was then added, and the mixture was incubated for a further 2 h at 4°C. Beads were washed three times in immunoprecipitation buffer, and the buffer was removed from the beads using a syringe. SDS sample buffer was added and the samples were heated for 5 min at 95°C before analysis by SDS-PAGE and Western blotting (Laemmli, 1970).

### SDS-PAGE and Western blotting

SDS-PAGE was performed using standard gels or with pre-cast 4–12% Bis-Tris gradient gels (Thermo Fisher Scientific). Gels were transferred by electrophoresis to nitrocellulose membranes using standard procedures (Towbin et al., 1979). In some cases, reversible Memcode protein staining (Thermo Fisher Scientific) was used to assess total protein content. Membranes were blocked for 1 h in 5% skim milk in PBS containing 1% Tween, and then probed using primary and secondary antibodies diluted in blocking buffer. Membranes were developed either using enhanced chemiluminescence or chemifluorescence. For chemiluminescence, photographic films were exposed for different times to obtain non-saturated chemiluminescence readouts. Alternatively, chemiluminescence was detected using a ChemoStar Touch ECL and Fluorescence Imager (Intas Science Imaging). Chemifluorescence was detected using an Odyssey Imaging System (LI-COR). Quantification was done using either the gels analysis function in ImageJ or the Odyssey image analysis software.

### Antibodies

Primary antibodies for Western blotting: Mouse anti-HA (1:1000, clone HA.11, BioLegend, 901,515, RRID: AB_2565334), rabbit anti-NACC1 (1:1000, Abcam, ab29047, RRID: AB_870608), rabbit anti-SynGAP1 (1:1000, Thermo Fisher Scientific, PA1-046, RRID: AB_2287112), rabbit anti-GluK2A/3 (1:800, clone NL9, Millipore, 04–921, RRID: AB_1587072). Secondary antibodies for Western blotting: HRP-conjugated goat anti-mouse (1:10000, Bio-Rad, 172–1,011, RRID: AB_11125936) and HRP-conjugated goat anti-rabbit (1:10000, Bio-Rad, 172–1,019, RRID: AB_11125143).

### Electrophysiology

Electrophysiological recordings in cultured neurons were performed as described (Burgalossi et al., 2012; Nair et al., 2013; Ripamonti et al., 2017). Autaptic neurons (11–13 DIV) were whole-cell voltage-clamped at −70 mV with an EPSC10 amplifier (HEKA) under the control of Patchmaster 2 (HEKA). Intracellular patch pipette solution consisted of (in mM): 136 KCl, 17.8 HEPES, 1 EGTA, 0.6 MgCl_2_, 4 NaATP, 0.3 Na_2_GTP, 15 creatine phosphate, and 5 U/mL phosphocreatine kinase (315–320 mOsmol/L, pH 7.4). Extracellular solution contained (in mM) 140 NaCl, 2.4 KCl, 10 HEPES, 10 glucose, 4 CaCl_2_ and 4 MgCl_2_ (320 mOsmL/L), pH, 7.3. On each day of recording, data were collected from each of the three conditions (EGFP, HA-Nacc1-WT, HA-Nacc1-R284W). Neurons for patch clamp recording were selected on the basis of exhibiting EGFP fluorescence and being isolated on a glial micro-island. Neurons were included in the analysis if they had an initial series resistance of less than 12 MΩ and exhibited a fast, autaptically evoked EPSC (eEPSC) typical of a glutamatergic neuron. eEPSCs were evoked by depolarizing the cell from −70 to 0 mV at 0.2 Hz. Ready releasable pool (RRP) size was measured by the application of 0.5 M hypertonic sucrose solution for 6 s using a fast-flowing micro-pipe. Vesicular release probability (P_vr_) was calculated by dividing the charge transfer during an evoked EPSC by the charge transfer during the sucrose response. Cell surface expression of glutamate receptors was assessed by focal application of 100 μM glutamic acid or 10 μM kainic acid using a fast-flowing micro-pipe. Miniature EPSCs (mEPSCs) were recorded for 100 s in the presence of 300 nM tetrodotoxin (TTX, Tocris Bioscience). All traces were analyzed using AxoGraph X (AxoGraph Scientific).

### Immunocytochemistry and synapse counting

All steps were performed at room temperature unless otherwise stated. In 6 well culture plates, with each well containing a coverslip of neurons, the coverslips were washed twice in PBS and then fixed for 12 min using 4% paraformaldehyde (in PBS). Cells were then washed 3x in PBS and then treated with 50 mM glycine (in PBS) for 10 min, and again washed 3x in PBS. A few drops of Image-iT™ FX Signal Enhancer (Thermo Fisher) was then added to each sample and the samples incubated for 5 min. Cells were then permeabilized and blocked by incubating for 45 min in 0.3% Triton X-100, 0.1% fish skin gelatin, and 10% normal goat serum (in PBS). Each coverslip was cut in half using a diamond knife, one half was incubated in anti-MAP2/anti-GFP/anti-HA/anti-Shank2 (to validate that HA-Nacc1 constructs and EGFP were successfully expressed in infected neurons) and the other half was incubated in anti-MAP2/anti-GFP/anti-VGLUT1/anti-Shank2 (for synapse counting). Coverslips were incubated at 4°C overnight. The following day, samples were washed 3 times in PBS, and fluorescent secondary antibodies applied for 60 min. Samples were then washed and mounted on glass slides using Aqua-Poly/Mount (Polysciences, USA). Primary antibodies were: chicken anti-MAP2 (1:2000, Novus Biologicals, catalog number: NB300-213, RRID: AB_2138178), mouse anti-GFP (1:2000, Roche, clones 7.1 and 13.1, catalog number: 11814460001, RRID: AB_390913), rabbit anti-GFP (1:2000, Abcam, catalog number: ab6556, RRID: AB_305564), mouse anti-HA (1:1000, Biolegend, catalog number: 901515, RRID: AB_2565334), rabbit anti-VGLUT1 (1:2000, Synaptic Systems, catalog number 135303, RRID: AB_887875), guinea pig anti-Shank2 (1:2000, Synaptic Systems, catalog number 162204, RRID: AB_2619861). Secondary antibodies were: Alexa Fluor 405 goat anti-chicken (1:1000, RRID: AB_2890271), Alexa Fluor 488 goat anti-mouse (1:2000, RRID: AB_2307324), Alexa Fluor 488 goat anti-rabbit (1:2000, RRID: AB_2630356), Alexa Fluor 568 goat anti-guinea pig (1:2000, RRID: AB_2534119), Alexa Fluor 633 goat anti-rabbit (1:2000, RRID: AB_2535732), Alexa Fluor 633 goat anti-mouse (1:2000, RRID: AB_2535719), all from Thermo Fisher. All antibodies were diluted in 2.5% normal goat serum (in PBS).

Imaging was performed using a Leica SP5 scanning confocal microscope, 40× oil immersion objective NA 1.25, 2048 × 2048 image resolution, 8 bit image depth. The generated data were analyzed for synapse counting by an in-house written macro in IJ1 for Fiji and ImageJ (NIH) in similar fashion to an earlier method (Rhee et al., 2019). The macro operates as follows. Presynaptic VGLUT1-positive structures are isolated by applying thresholds, which are converted to masks, and then selections are created. As synapses form on dendrites, which are positive for MAP2, VGLUT1-positive selections that do not coincide with the MAP2-positive area are eliminated. The remaining VGLUT1-positive selections are then multiplied in size by 1 pixel. This process is then also performed for anti-Shank2 immunolabeling, but without the selection enlargement step. Finally, puncta from the VLGUT1 and Shank2 selections that co-localized are counted as synapses.

### Data structure and statistical analysis

Statistical analyses were performed using Prism 8 software (GraphPad). No randomization methods were used to allocate samples in this study. No group allocation, randomization, pre-registration or power calculations were performed. This study was exploratory and no primary or secondary endpoints were pre-specified. All experiments were performed at least 3 times. For most experiments, data were defined as statistically independent if they were derived from separate experiments (in the case of experiments using cultured cell lines) or separate animals in the case of primary neuronal cultures. In the case of synapse counting, this experiment was performed using one coverslip of neurons per condition, taken from a single neuronal culture. In this case, the number of cells analyzed was used as the sample size, *n*.

For electrophysiology, individual neurons were defined as statistically independent for the purposes of analysis. As regards the structure of electrophysiology data, for each round of experiments a single batch of neurons were generated from one neonatal mouse. These neurons were then infected at DIV 1 with either of the 3 viruses described above, with an equal number of cell culture wells infected with each virus, so that there were three treatment groups, EGFP, Nacc1-WT, and Nacc1-R284W. Neurons were then allowed to mature, and electrophysiology experiments were conducted on subsequent days, starting on day 11 and finishing on day 13 after neuron preparation. Data were collected from at least four separate batches of neurons. To examine whether the neurons exhibited significant functional differences either between the different ages examined (DIV 11, 12, 13) or between batches of cells (i.e., between animals, since each batch of cells came from a different animal), we used ANOVA to examine whether the eEPSC amplitudes varied when data were grouped according to age or cell culture batch. eEPSC amplitude was used as a general metric of cell functionality. eEPSC amplitude did not vary significantly with the age of the cells, and thus cells from all ages were pooled regardless of age. However, one batch of wild-type neurons exhibited significantly lower eEPSC amplitudes compared to other culture batches, and thus data from that neuron batch was excluded from analysis. Data for each treatment group were then pooled regardless of cell batch and cell age. Data were assessed for normality of distribution using the Shapiro-Wilks normality test. To analyze data for statistically significant differences, data were compared using tests as described in the figure legends. For all figures, * denotes a *p* value of between 0.01 and 0.05, ** denotes a *p* value of between 0.001 and 0.01, and *** denotes a *p* value of between 0.0001 and 0.001. In data from electrophysiology experiments, only statistical comparisons yielding significant differences are denoted on graphs, non-significant comparisons are not denoted.

## Results

### Expression of Nacc1-R284W in *Nacc1^+/−^* neurons impairs excitatory synaptic transmission

**Figure 1:**
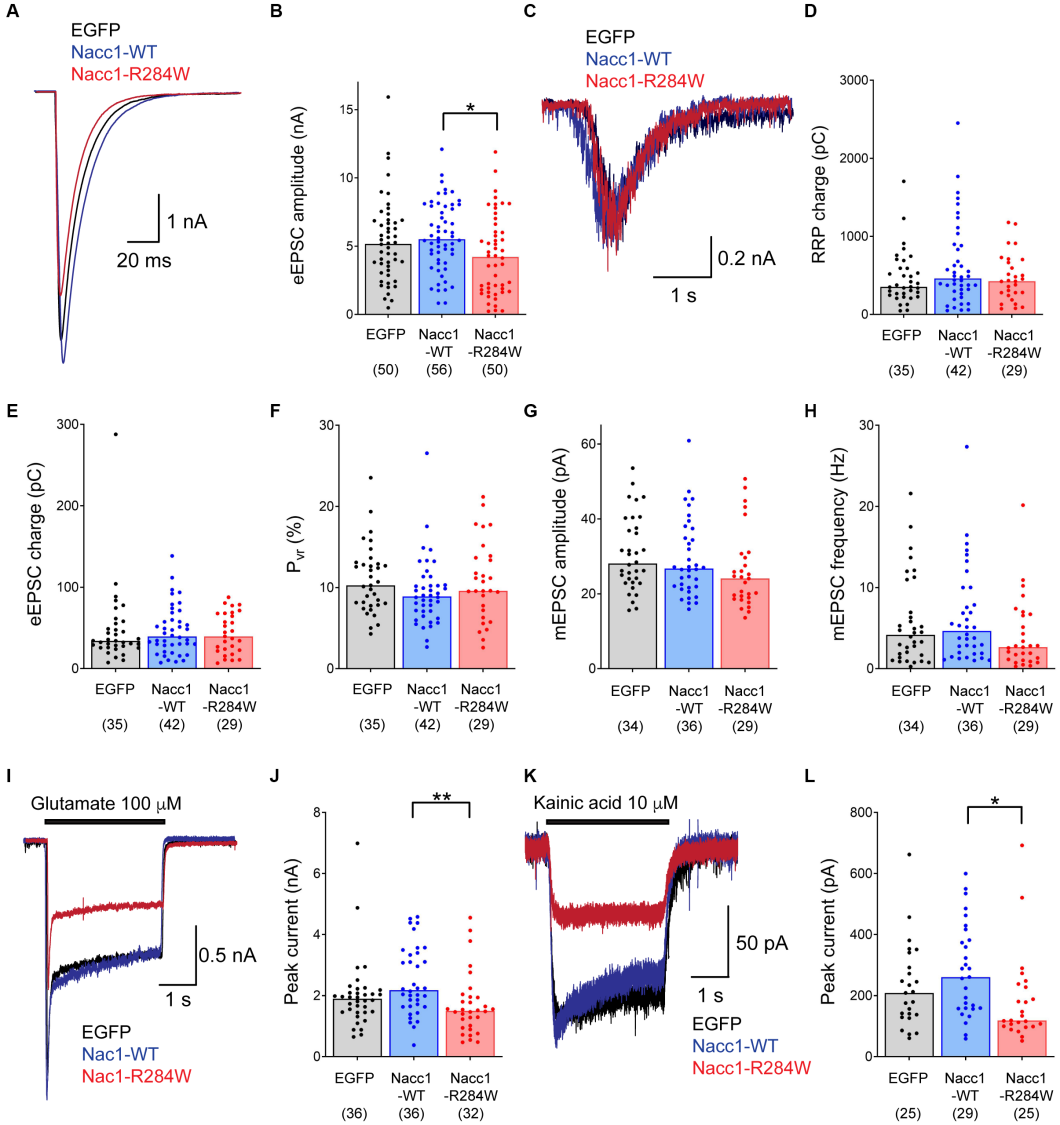
Expression of Nacc1-R284W decreases EPSC amplitude compared to Nacc1-WT in *Nacc1^+/−^* glutamatergic neurons. Autaptic glutamatergic hippocampal neurons from *Nacc1^+/−^* mice were transduced with lentivirus inducing expression of either EGFP (black), Nacc1-WT (blue), or Nacc1-R284W (red). Synaptic function was then examined using patch clamp recording. **(A)** Overlaid representative eEPSC traces taken from a single cell from each transduction condition. **(B)** Bar charts showing the eEPSC amplitudes measured from single autaptic neurons transduced as above. **(C)** Representative overlaid traces of the current evoked by the application of 500 mM sucrose to evoke fusion of synaptic vesicles (SVs) in the RRP. **(D)** Bar charts showing the average total charge transferred by the release of the RRP in individual neurons transduced as above. **(E)** Bar charts showing the charge transferred by the eEPSC in individual neurons. **(F)** Bar charts showing the probability of vesicular release (P_vr_) of individual neurons transduced as above. P_vr_ was calculated by dividing the charge transfer during an evoked EPSC by the charge transfer during the sucrose response, and then expressed as a percentage. **(G)** Bar charts showing the amplitude of spontaneous miniature EPSCs (mEPSCs) recorded from individual transduced neurons in the presence of 300 nM TTX. **(H)** Bar charts showing the frequency (Hz) of mEPSCs recorded from individual transduced neurons. **(I)** Overlaid representative traces of the current induced by perfusion with 100 μM glutamate. **(J)** Bar charts showing the amplitude of the peak current generated by glutamate application in transduced neurons. **(K)** Overlaid representative traces of the current induced by perfusion with 10 μM kainic acid (KA). **(L)** Bar charts showing the amplitude of the peak current generated by KA application in transduced neurons. In each chart, dots represent values recorded for individual cells, while the bar height represents the median of all cells. The number of cells analyzed for each condition is displayed beneath the condition label on each charts. Data were compared using a Kruskal-Wallis test by ranks followed by Dunn’s multiple comparisons test. * Denotes a *p* value of between 0.01 and 0.05, ** denotes a *p* value of between 0.001 and 0.01.

Heterozygous expression of a *de novo* NACC1-R298W variant causes profound defects in neural development and function in human patients (Schoch et al., 2017), but the mechanisms by which this phenotype arises are unclear. As intellectual disability is associated with disruption of synaptic transmission (Hamdan et al., 2011; Parker et al., 2015), we examined excitatory synaptic function in neurons expressing Nacc1-R284W, the mouse equivalent of NACC1-R298W (Figure 1; Supplementary Figure S1A). To mimic the heterozygous context in patients, we used Nacc1^+/−^ autaptic hippocampal neurons, which exhibit 50% lower Nacc1 levels compared to WT (*NACC1^+/+^*) mice (Supplementary Figures S2A,B).

*Nacc1^+/−^* neurons expressing Nacc1-R284W displayed a moderate reduction in the amplitude of evoked excitatory post-synaptic currents (eEPSC) compared to *Nacc1^+/−^* neurons expressing Nacc1-WT or EGFP (Figures 1A,B). Surprisingly however, detailed analyses of presynaptic function did not detect any further changes in the size of the readily-releasable pool (RRP) of synaptic vesicles, EPSC charge, released probability (P_vr_), or miniature EPSC (mEPSC) frequency and amplitude in *Nacc1^+/−^* neurons expressing Nacc1-R284W compared to neurons expressing either EGFP or Nacc1-WT (Figures 1C–H). A statistically non-significant trend toward a reduction in mEPSC frequency in neurons expressing Nacc1-R284W was observed (Figure 1H), possibly indicative of a reduction in synapse number. However, while a small decrease in membrane capacitance was observed in *Nacc1^+/−^* neurons expressing Nacc1-R284W as compared to neurons expressing Nacc1-WT (Supplementary Figure S2C), immunocytochemical analysis of the number of synapses formed by each neuron showed no alterations in synapse numbers between neurons expressing EGFP, Nacc1-WT, or Nacc1-R284W (Supplementary Figure S3). Overall, these data indicate that expression of Nacc1-R284W impairs glutamatergic neurotransmission in *Nacc1^+/−^* neurons, but that this impairment is not the result of altered presynaptic function.

Therefore, we investigated changes in post-synaptic function in more detail, focusing on glutamatergic neurotransmission. We evoked glutamate receptor-mediated currents independently of presynaptic glutamate release by briefly superfusing infected neurons with 100 μM glutamate during patch clamp recording. Neurons expressing Nacc1-R284W exhibited significantly reduced glutamate-induced currents compared to neurons expressing Nacc1-WT (Figures 1I,J), indicating that expression of Nacc1-R284W causes reduced levels of glutamate receptors on the neuronal surface.

The current evoked by exogenously applied glutamate is primarily mediated by AMPA receptors. Patients expressing NACC1-R298W exhibit severe epilepsy (Schoch et al., 2017). Glutamate receptors of the kainic acid (KA) type have long been implicated in epilepsy. Exogenous KA administration induces seizures through the activation of KA receptors, and is a key rodent model of epilepsy (Mulle et al., 1998; Crepel and Mulle, 2015). Therefore, we examined KA-mediated glutamatergic neurotransmission more specifically. Strikingly, superfusion of *Nacc1^+/−^* neurons expressing Nacc1-R284W with 10 μM KA elicited significantly smaller currents than neurons expressing Nacc1-WT (Figures 1K,L), indicating reduced levels of KA receptors on the neuronal surface. Interestingly, NMDAergic currents were unaltered (Supplementary Figure S2D).

Overall, these data demonstrate that expression of the disease-associated Nacc1 mutant Nacc1-R284W in *Nacc1^+/−^* neurons causes decreased EPSC amplitude, along with decreased responsiveness to exogenously applied glutamate and KA. These data indicate that glutamate receptor expression at the neuronal surface is compromised by the expression of Nacc1-R284W.

### Expression level of endogenous Nacc1-WT determines the impact of Nacc1-R284W on synaptic function

Genetic deletion of Nacc1 has no major neurological or developmental consequences (Yap et al., 2013). Accordingly, electrophysiological analyses of Nacc1-KO (i.e., *Nacc1^−/−^*) neurons only revealed a small reduction in P_vr_ compared to WT neurons from the same litter (Supplementary Figure S4D), with no changes in other basic features of glutamatergic synapse function (Supplementary Figures S4A–C,E–G). Given that Nacc1 deletion in neurons does not induce changes in glutamatergic neurotransmission analogous to those seen in *Nacc1^+/−^* neurons expressing Nacc1-R284W, it is likely that Nacc1-R284W is a dominant negative rather than a loss of function variant. This also implies that the levels of endogenous Nacc1-WT expressed might determine the impact of Nacc1-R284W on synaptic function.

To examine this hypothesis, we expressed WT or Nacc1-R284W in WT *Nacc1^+/+^* neurons (Supplementary Figure S5). As with *Nacc1^+/−^* neurons, *Nacc1^+/+^* neurons expressing Nacc1-R284W exhibited reduced eEPSC amplitudes (Supplementary Figures S5A,B) and a diminished response to exogenously applied glutamate (Supplementary Figures S5C,D). These observations indicate that the impact of Nacc1-R284W expression on the eEPSC amplitude and glutamate-evoked current is similar in *Nacc1^+/−^* neurons and WT neurons. Strikingly, however, no significant change in KA-induced current was observed in *Nacc1^+/+^* neurons expressing Nacc1-R284W (Supplementary Figures S5E,F), in contrast to the substantial reduction in KA-evoked current observed in *Nacc1^+/−^* neurons expressing Nacc1-R284W (Figures 1K,L). As *Nacc1^+/−^* neurons express half the amount of Nacc1 compared to *Nacc1^+/+^* neurons (Supplementary Figure S2), these data indicate that the expression level of endogenous Nacc1 can mitigate the impact of Nacc1-R284 on KA receptor function in neurons. Further, like in *Nacc1^+/−^* neurons, mEPSC properties were unaltered upon expression of Nacc1-R284W in *Nacc1^+/+^* neurons (Supplementary Figures S5G,H).

Surprisingly, *Nacc1^+/+^* neurons expressing Nacc1-R284W exhibited decreased RRP charge, decreased eEPSC charge, and increased P_vr_ compared to *Nacc1^+/+^* neurons expressing Nacc1-WT (Supplementary Figures S5I–L). These data indicate presynaptic dysfunction in *Nacc1^+/+^* neurons expressing Nacc1-R284W, which was not observed in *Nacc1^+/−^* neurons expressing Nacc1-R284W (Figure 1F).

Overall, these findings complement our observations in *Nacc1^+/−^* neurons that Nacc1-R284W expression results in impaired glutamatergic neurotransmission, with the surprising finding that Nacc1-R284W impairs presynaptic function in *Nacc1^+/+^* neurons. Furthermore, these data demonstrate that the level of endogenous Nacc1 expression influences the severity of the synaptic dysfunction caused by Nacc1-R284W expression.

### Nacc1 binds to the synaptic proteins SynGAP1 and GluK2A

No proteins directly associated with glutamatergic neurotransmission have been identified as Nacc1 binding partners to date. Therefore, to identify novel Nacc1 interaction partners of potential relevance in neuronal function, we performed a yeast two-hybrid (Y2H) screen, employing a postnatal day 8 rat brain cDNA library as bait and full-length Nacc1-WT as prey. This yielded a ‘validated’ collection of 26 clones encoding 16 novel Nacc1 interactor candidates (Table 1; Supplementary Figure S6). Interestingly, we identified the synaptic Ras GTPase activating protein 1, SynGAP1, as a potential Nacc1 interaction partner (Jeyabalan and Clement, 2016).

**Table 1:** List of validated Nacc1 interaction partners.

Protein name	Abbreviation	# of Clones	
		Unique	Total
Zinc finger MYM-type 2	Zmym2	4	4
Protein inhibitor of activated STAT 2*	Pias2	3	12
Ring Finger protein 111*	RNF111	2	25
Nucleus acumbens associated protein 1	Nacc1	2	4
Zinc finger and BTB domain 18*	zBTB18	2	4
L3MBTL2 polycomb repressive complex 1 subunit	L3MBTL2	2	2
Synaptic Ras GTPase activating protein 1*	SynGAP1	2	2
CCHC domain containing 18*	Zcchc18	1	10
Protein inhibitor of activated STAT 1*	Pias1	1	3
Caspase 8 associated protein 2*	casp8ap2	1	2
Protein inhibitor of activated STAT 3*	Pias3	1	1
Polo-like kinase 2*	Plk2	1	1
RAN binding protein 2*	Ranbp2	1	1
Histone acetyltransferase KAT5*	Kat5	1	1
Activating transcription factor 7-interacting protein*	Atf7ip	1	1
G-protein coupled receptor associated sorting 1*	gprasp1	1	1

Names of the prey clones identified and validated in the Nacc1 yeast two-hybrid screen. Previously unreported interaction partners are indicated (*). All prey fragments of the listed proteins were positive for both reporter genes (HIS3 and LacZ). The identity of these proteins was determined using the Basic Local Alignment Search tool (BLAST) and the GenBank database (NCBI). The number of total clones indicates the number of prey fragments isolated in total, while the number of unique clones indicates the number of prey clones that encoded distinct sequences within the protein.

**Figure 2:**
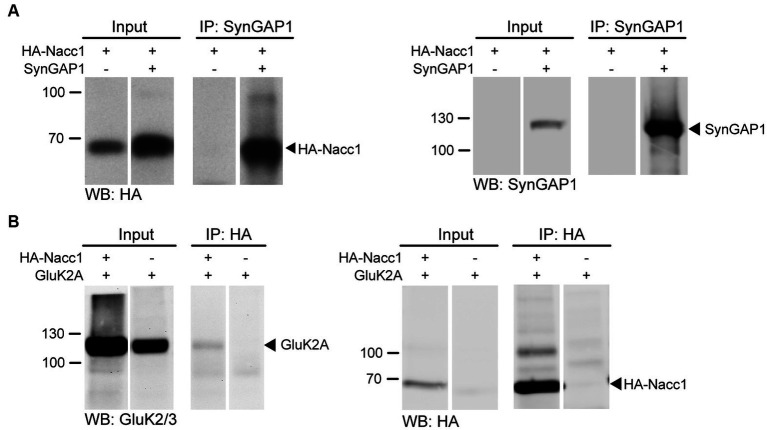
Nacc1 interacts with the synaptic proteins SynGAP1 and GluK2A. **(A)** Anti-HA (left panel) and anti-SynGAP1 (right panel) Western blot analysis of input and eluate fractions from an anti-SynGAP1 affinity purification (IP: anti-SynGAP1) in N2A cells expressing SynGAP1 and HA-Nacc1 alone or in combination. Black arrowheads indicate unmodified HA-Nacc1 (left) or SynGAP1 (right). **(B)** Anti-GluK2A/3 (left panel) and anti-HA (right panel) Western blot analysis of input and eluate fractions from an anti-HA affinity purification (IP: anti-HA) in N2A cells expressing GluK2A and HA-Nacc1 alone or in combination. A black arrowhead indicates GluK2A in the left blot. On the right, a black arrowhead indicates unmodified HA-Nacc1. Molecular weight markers (kDa) are indicated to the left of the blots. Individual lanes of single Western blot membranes are shown in **A** and **B**, since several lanes contained samples not relevant to the figure. The full blots are available in Supplementary Figure S8. Images are representative of at least 3 independent experiments.

To confirm a direct interaction between Nacc1 and SynGAP1, we performed anti-SynGAP1 immunoprecipitation from mammalian N2A cells overexpressing HA-Nacc1 and SynGAP1 (Figure 2). Western blot analysis of input and immunoprecipitated fractions confirmed enrichment of HA-Nacc1 from cell lysates by anti-SynGAP1 immunoprecipitation (Figure 2A, black arrowhead), indicating that overexpressed Nacc1 and SynGAP1 interact in mammalian cells.

Although the KA receptor subunit GluK2A did not appear in the Y2H screen, we tested whether Nacc1 interacts with GluK2A, because (i) ‘heterozygous’ expression of Nacc1-R284W impaired KA-induced current (Figure 1) and (ii) GluK2A is ubiquitinated by both cullin-3 and parkin, which are also binding partners of Nacc1 (Shen et al., 2007; Korutla et al., 2014). We transfected cells with HA-Nacc1 and GluK2A and found that affinity purification of overexpressed HA-Nacc1 indeed resulted in co-purification of GluK2A, confirming an interaction between the two proteins (Figure 2B, black arrowhead).

Overall, these data identify a range of novel Nacc1 interactor candidates and suggest that, *in vitro*, Nacc1 interacts with SynGAP1 and GluK2A, both components of the postsynaptic machinery at glutamatergic synapses. *In vivo*, all three proteins of interest, i.e., Nacc1, GluK2A and SynGAP1, are found in crude P2 synaptosomes and in the S2 synaptic cytosol fractions from mouse brain (Supplementary Figure S2E), where the proteins could interact with one another.

### Nacc1 is SUMOylated

Interestingly, the Y2H screen identified four SUMO E3 ligases as binding partners of Nacc1 (PIAS 1–3 and RanBP2, see Table 1; Supplementary Figure S6). Using an overexpression cell model followed by co-immunoprecipitation, we confirmed the interaction of Nacc1-WT with one of the PIAS family members, PIAS2 (Supplementary Figure S7A). In addition, enrichment of the disease-associated mutant Nacc1-R284W also resulted in co-immunoprecipitation (co-IP) of PIAS2, indicating that this mutant also binds to PIAS2 (Supplementary Figure S7B). Therefore, we further characterized the SUMOylation of Nacc1 *in vivo* and *in vitro*.

**Figure 3:**
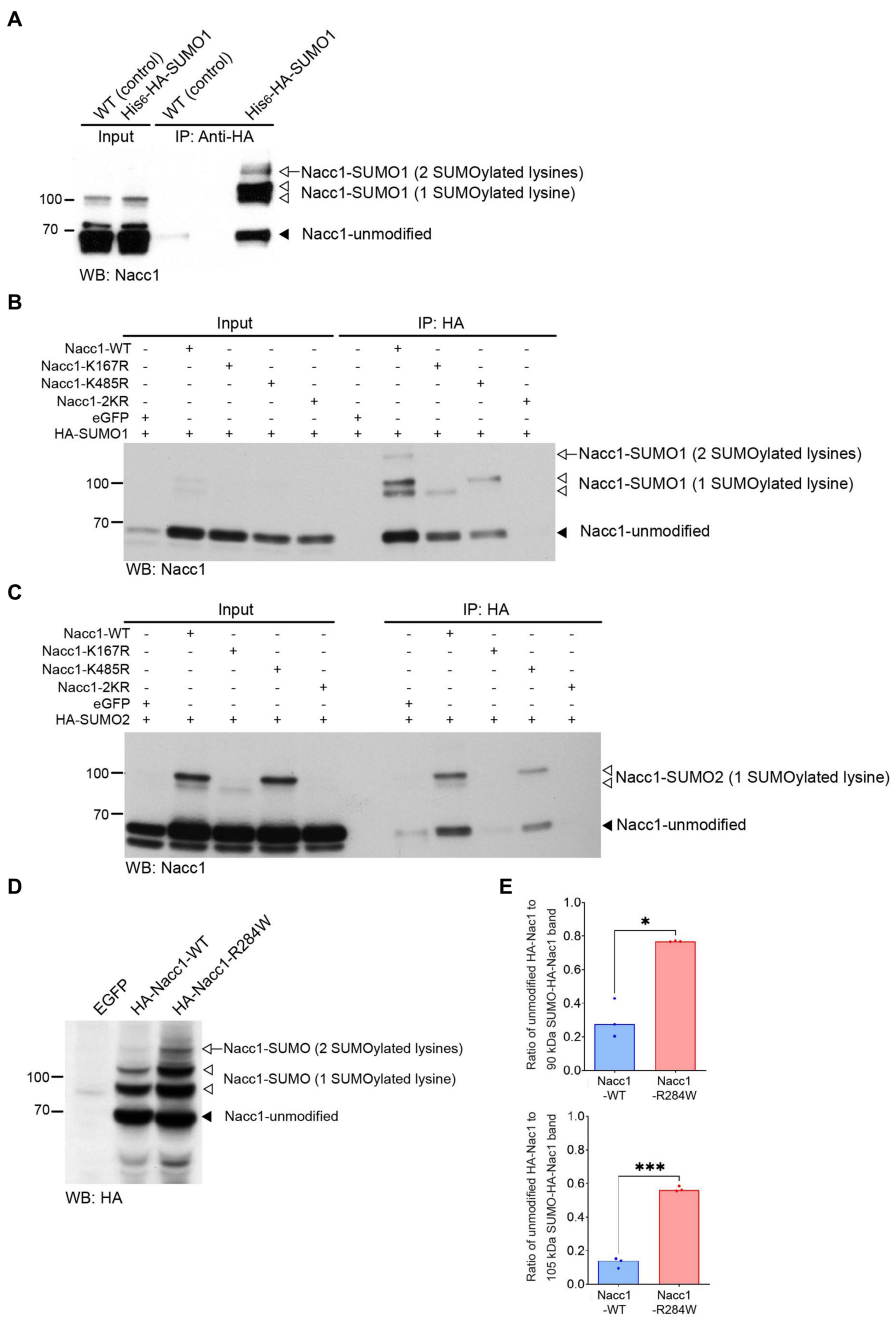
Nacc1 is conjugated to SUMO *in vivo* and *in vitro* and Nacc1-R284W exhibits increased SUMO conjugation in neurons. **(A)** Western blot analysis using anti-Nacc1 antibody of input and HA peptide eluate fractions from an anti-HA affinity purification (IP: anti-HA) of WT (WT) and His_6_-HA-SUMO1 knock-in (KI) mouse brain. The black arrowhead indicates the position of non-modified Nacc1, the white arrowhead indicates Nacc1 modified with one SUMO1 peptide (appearing as doublet), and the white arrow indicate Nacc1 modified with two SUMO1 peptides. **(B)** Anti-Nacc1 Western blot analysis of input and eluate fractions from an anti-HA affinity purification (IP: anti-HA) in N2A cells expressing HA-SUMO1 plus either EGFP, Nacc1-WT, Nacc1-K167R, Nacc1-K485R, or Nacc1-2KR. The black arrowhead indicates unmodified Nacc1, the white arrowheads indicate Nacc1 conjugated to a single SUMO1 peptide (appearing as a doublet), and the white arrow indicates Nacc1 conjugated to two SUMO1 peptides. **(C)** Anti-Nacc1 Western blot analysis of input and affinity purification eluate fractions (IP: anti-HA) of N2A cells expressing HA-SUMO2 plus either EGFP, Nacc1-WT, Nacc1-K167R, Nacc1-K485R, or Nacc1-2KR. The black arrowhead indicates unmodified Nacc1 and the white arrowheads indicate Nacc1 conjugated to a single SUMO2 peptide (appearing as a doublet). Molecular weight markers (kDa) are indicated to the left of the blots. **(D)** Anti-HA Western blot analysis of lysate from primary hippocampal neuron infected with lentivirus mediating expression of EGFP, HA-Nacc1-WT, or HA-Nacc1-R284W. The black arrowhead indicates non-modified Nacc1, the white arrowheads indicate Nacc1 modified with one SUMO peptide, and the white arrow indicates Nacc1 modified with two SUMO peptides. **(E)** Bar charts showing a quantification of the ratio of unmodified Nacc1 to the SUMO-Nacc1 band at 90 kDa (upper chart), or to the SUMO-Nacc1 band at 105 kDa (lower chart). Data were normalized to the total protein loading for each well. Data were compared using a Welch *t*-test. * Denotes a *p* value of between 0.01 and 0.05, *** denotes a *p* value of between 0.0001 and 0.001. Images are representative of at least 3 independent experiments. Corresponding anti-HA blots for the experiments shown in **(B)** and **(C)** are presented in Supplementary Figure S9. Molecular weights in kDa are indicated next to all blots.

Nacc1 was identified as a SUMOylation target in proteomics screens conducted in mammalian cell lines and mouse brain tissue (Schou et al., 2014; Hendriks et al., 2015, 2018; Tatemichi et al., 2015). To further examine Nacc1 SUMOylation *in vivo*, we focused on SUMO1 and enriched SUMO1-conjugated proteins from the brains of His_6_-HA-SUMO1 knock-in mice by anti-HA immunopurification (Tirard et al., 2012; Tirard and Brose, 2016; Daniel et al., 2017, 2018; Stankova et al., 2018; Ripamonti et al., 2020). Western blot analysis using anti-Nacc1 antibodies showed a major Nacc1 band at 65 kDa in input samples from WT and His_6_-HA-SUMO1 mice (Figure 3A, black arrowhead), representing unmodified Nacc1. In the input samples a doublet of Nacc1-positive bands was apparent at 90/105 kDa (Figure 3A, white arrowheads), possibly representing SUMOylated forms of Nacc1. Anti-HA immunoprecipitation (IP) in samples from His_6_-HA-SUMO1 mice resulted in an enrichment of the Nacc1-positive bands at 90/105 kDa (Figure 3A, white arrowheads), along with a Nacc1-positive band at 120 kDa (Figure 3A, white arrow). These enriched bands were absent in control samples from WT mice (Figure 3A). The presence of unSUMOylated Nacc1 in the anti-HA IP eluate from KI mice either indicate non-specific binding of Nacc1 to the beads (as revealed by a weak signal observed in the eluate from WT), and/or dimerization between SUMOylated and SUMOylated Nacc1 proteins. Overall, these data indicated that Nacc1 is conjugated to SUMO1 *in vivo*.

K167 and K485 of mouse Nacc1 were previously proposed as SUMOylation sites (Hendriks et al., 2018). To test this, we generated three ‘SUMOylation-deficient’ Nacc1 mutants in which K167 and K485 were mutated to arginines, Nacc1-K167R, Nacc1-K485R and Nacc1-K167R/K485R (referred to as Nacc1-2KR, Supplementary Figure S1B). N2A cells were co-transfected with these mutants plus either HA-SUMO1 (Figure 3B) or HA-SUMO2 (Figure 3C). This was then followed by anti-HA IP to enrich SUMO-conjugated proteins and then immunoblotted with anti-Nacc1. Western blotting analysis demonstrated that mutation of K167 abolished the Nacc1-positive bands at 105 and 120 kDa in the IP fraction (Figure 3B, top white arrowhead and white arrow), whereas mutation of K485 abolished the bands at 90 and 120 kDa (Figure 3B, bottom white arrowhead and white arrow). Mutation of K167 and K485 together abolished the Nacc1 bands at 90, 105 and 120 kDa, as no Nacc1 positive bands was detected in the IP fraction (Figure 3B). Taken together, these data indicated that the bands at 90 and 105 kDa represented Nacc1 mono-SUMO1ylated at a single residue, with the two forms of mono-SUMOylated Nacc1 running at different apparent molecular weights (Figures 3B,C). Additionally, the Nacc1 band at 120 kDa represents Nacc1 that is mono-SUMO1ylated at both K167 and K485 (Figures 3B,C). Importantly, the Nacc1-positive bands at 90, 105 and 120 kDa in transfected N2A cells (Figures 3B,C) correspond with the Nacc1-positive bands enriched by anti-HA IP in brain homogenates from His_6_-HA-SUMO1 mice. In the case of SUMO2, K167 almost fully abolished Nacc1 SUMOylation, as no shifted bands were detected after IP (Figure 3C), while K485R led to the disappearance of the Nacc1 band at 90 kDa (Figure 3C, bottom white arrowhead). Mutation of K167 and K485 together abolished the Nacc1 bands at 90 and 105, as no Nacc1 positive bands was detected in the IP fraction (Figure 3C). Overall, these data confirm that Nacc1 can be SUMOylated *in vivo* and *in vitro* by either SUMO1 or SUMO2 and that K167 and K485 are the only SUMO-acceptor lysines.

### The R284W mutation causes increased SUMO conjugation of Nacc1 in neurons

We next investigated whether Nacc1-R284W is subject to SUMOylation in neurons. Hippocampal neurons were transduced with lentiviruses encoding either EGFP alone or EGFP with HA-Nacc1-WT or HA-Nacc1-R284W. Western blot analysis of total protein extracts from infected neuron cultures showed that HA-Nacc1 constructs were SUMO-conjugated in cultured neurons, and that SUMOylation was strikingly increased for HA-Nacc1-R284W compared to HA-Nacc1-WT (Figure 3D, see bands indicated by the white arrow and white arrowheads). Strikingly, quantitative analysis of the intensity of the HA-positive bands at 90 kDa and 105 kDa (SUMO-conjugated Nacc1) relative to the unmodified Nacc1 (65 kDa) in each sample revealed that both the 90 kDa and 105 kDa bands were more abundant for HA-Nacc1-R284W compared to HA-Nacc1-WT (Figure 3E). In addition, HA-Nacc1-R284W exhibited higher molecular weight bands not observed in HA-Nacc1-WT samples (Figure 3E), likely indicating poly-SUMOylated Nacc1. Overall, these data show that the R284W mutation causes increased SUMOylation of Nacc1.

### Nacc1-R284W exhibits reduced binding to SynGAP1 and GluK2A

**Figure 4:**
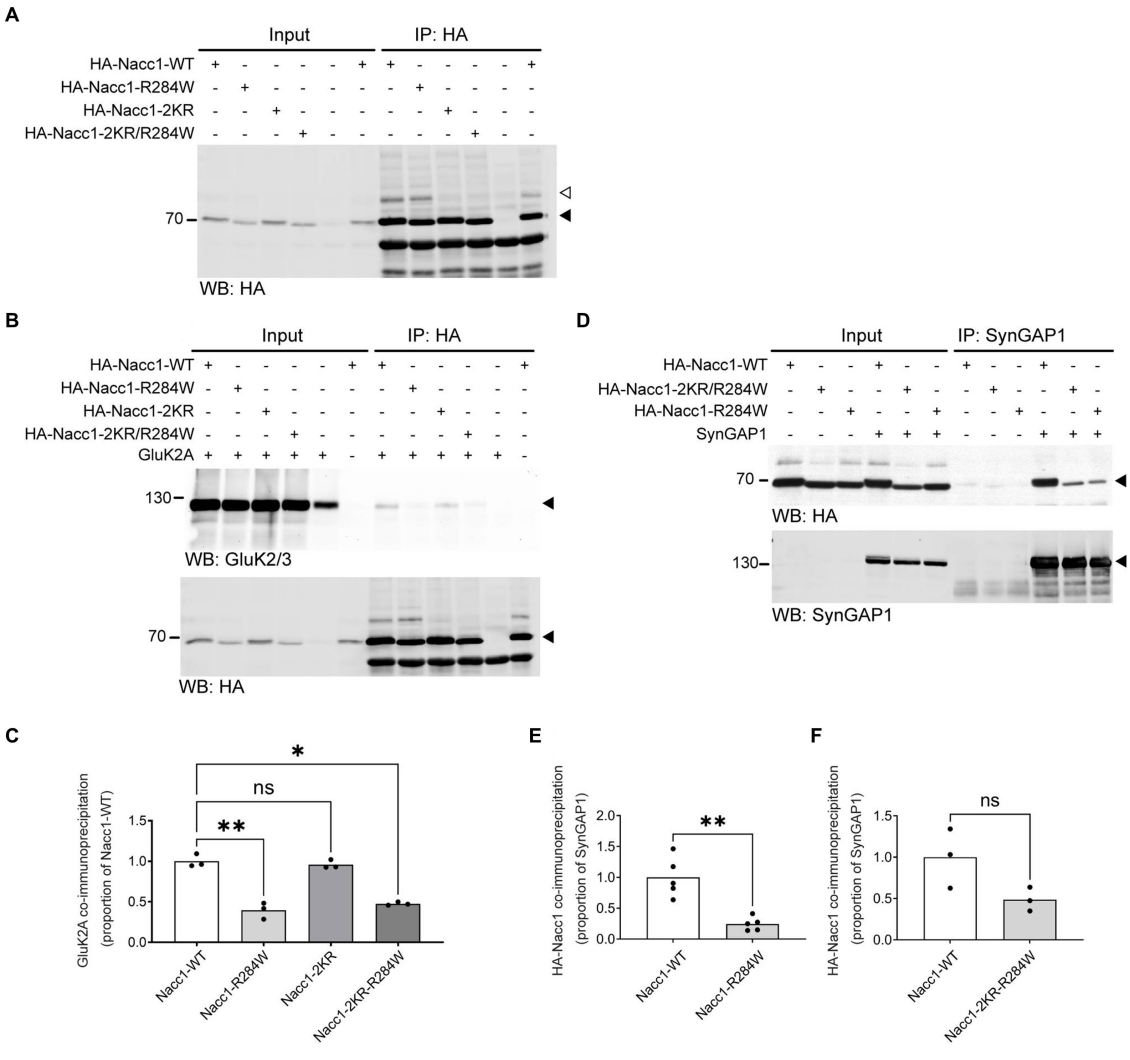
Nacc1-R284W exhibits impaired binding to SynGAP1 and GluK2A. **(A)** Representative anti-HA Western blot analysis of input and eluate fractions from an anti-HA affinity purification (IP: anti-HA) in N2A cells expressing one of four HA-Nacc1 constructs, as indicated above the blot. Black arrowhead indicates unmodified Nacc1, white arrowhead indicates Nacc1 conjugated to a SUMO protein. **(B)** Anti-GluK2A (top) and anti-HA (bottom) Western blot analysis of input and eluate fractions from an anti-HA affinity purification (IP: anti-HA) in N2A cells expressing GluK2A alone or in combination with one of four HA-Nacc1 constructs as indicated on top of the blot/images. A black arrowhead indicates GluK2A (top panel) or non-SUMO modified Nacc1 (bottom panel). **(C)** Bar charts showing quantification of the amount of GluK2A enriched after anti-HA-Nacc1 affinity purification using the various HA-Nacc1 mutants. The amount of GluK2A present in the eluate sample after anti-HA affinity purification was normalized firstly to the amount of GluK2A in the corresponding input sample, then to the amount of HA-Nacc1 enriched in the corresponding sample after affinity purification, and then finally expressed as a proportion of the amount of enrichment observed in the HA-Nacc1-WT sample. Data were compared using a Brown-Forsythe ANOVA followed by a Dunnett’s T3 multiple comparisons test. * Denotes a *p* value of between 0.01 and 0.05, ** denotes a *p* value of between 0.001 and 0.01. In each chart, dots represent values for a biological replicate and the bar height represents the median of all cells. *N* = 3 independent experiments. **(D)** Anti-HA (top panel) and anti-SynGAP1 (bottom panel) Western blot analysis of input and eluate fractions from an anti-SynGAP1 affinity purification (IP: anti-SynGAP1) of N2A cells expressing SynGAP1 alone or in combination with the various Nacc1 constructs as indicated on top of the blot. A black arrowhead indicates unmodified Nacc1 (top panel) or SynGAP1 (bottom panel). **(E)** Bar chart showing quantification of the amount of HA-Nacc1-R284W enriched by anti-SynGAP1 affinity purification, compared to HA-Nacc1-WT. The amount of each HA-Nacc1 construct present in the eluate sample after anti-SynGAP1 affinity purification was normalized firstly to the amount of HA-Nacc1 in the corresponding input sample, then to the amount of SynGAP1 enriched in the corresponding sample after affinity purification, and then finally expressed as a proportion of the amount of enrichment observed in the HA-Nacc1-WT sample. In each chart, dots represent values for a biological replicate and the bar height represents the median of all cells. Data were compared using a Welch’s *t*-test. ** Denotes a *p* value of between 0.001 and 0.01. *N* = 5 independent experiments. **(F)** Bar chart showing quantification of the amount of Nacc1-R284W-2KR enriched by anti-SynGAP1 affinity purification, compared to Nacc1-WT. Data normalization was performed as described for **(E)**. In each chart, dots represent values for a biological replicate and the bar height represents the median of all cells. NS denotes no significant difference. *N* = 3 independent experiments. Molecular weights in kDa are indicated next to all blots.

We next examined the impact of the R284W mutation on the ability of Nacc1 to interact with other proteins. Importantly, mutation of both SUMO acceptor lysines in Nacc1-R284W abolished SUMOylation of the protein (HA-Nacc1-2KR-R284W, Figure 4A, white arrowhead).

We then analyzed the co-immunoprecipitation of GluK2A by each of these HA-Nacc1 constructs (Figure 4B). Co-immunoprecipitation of GluK2A was significantly reduced (by 60%) after HA immunopurification from cells overexpressing HA-Nacc1-R284W as compared to HA-Nacc1-WT (Figure 4C). Cells overexpressing HA-Nacc1-2KR exhibited normal levels of GluK2A co-immunoprecipitation, thus indicating that SUMOylation is not required for Nacc1-GluK2A interaction. To examine whether increased SUMOylation of HA-Nacc1-R284W (Figures 3D,E) causes decreased binding to GluK2A, we examined the co-immunoprecipitation of GluK2A by the construct HA-Nacc1-2KR-R284W. Co-immunoprecipitation of GluK2A was significantly reduced (by 52%) in cells overexpressing HA-Nacc1-R284W as compared to HA-Nacc1-WT (Figure 4C), indicating that hyper-SUMOylation of HA-Nacc1-R284W is not the cause of reduced binding between Nacc1-R284W and GluK2A.

Similarly, we also examined whether the R284W mutation disrupts Nacc1 interaction with SynGAP1. Overexpression of the various Nacc1 constructs along with SynGAP1 followed by anti-SynGAP1 affinity purification revealed that the R284W mutation decreases Nacc1 interaction with SynGAP1, as seen with GluK2A (Figure 4D). Compared to HA-Nacc1-WT, significantly less HA-Nacc1-R284W was co-immunoprecipitated following anti-SynGAP1 immunoprecipitation, with an overall reduction in co-immunoprecipitation of 75% (Figure 4E). Strikingly, HA-Nacc1-2KR-R284W did not exhibit a significant reduction in binding to SynGAP1 (Figure 4F), in contrast with HA-Nacc1-R284W, indicating that the SUMOylation sites of Nacc1-R284W are important in binding to SynGAP1. Thus, hyper-SUMOylation may alter Nacc1-R284W binding to SynGAP1, in contrast to our observations with GluK2A (Figure 4C).

## Discussion

The identification of a *de novo* disease-associated NACC1 mutant (NM_052876.3:g.892C>T, NP_443108.1;p.Arg298Trp, referred to here as NACC1-R298W for the human mutant and as Nacc1-R284W for the murine mutant) in human patients with multiple neurological defects led us to examine the functional impact of this mutation on glutamatergic neurotransmission. We report that exogenous expression of Nacc1-R284W, the murine homolog of NACC1-R298W, impairs glutamatergic synaptic transmission in a dominant negative manner. We further found that the R284W mutation causes striking biochemical changes in Nacc1, including loss of binding to key synaptic proteins and increased SUMOylation.

### Nacc1-R284W perturbs glutamatergic neurotransmission

In *Nacc1^+/−^* neurons, the expression of Nacc1-R284W decreased eEPSC amplitudes, demonstrating that Nacc1-R284W expression inhibits evoked glutamatergic synaptic transmission. In addition, Nacc1-R284W expression decreased the peak currents elicited by the exogenous application of glutamate and KA, which activate both synaptic and extra-synaptic glutamate receptors. However, unaltered mEPSC amplitude in cells expressing Nacc1-R284W indicates that synaptic glutamatergic receptors are not specifically affected. Therefore, these data indicate a reduction of total cell-surface AMPA and KA receptor levels in cells expressing Nacc1-R284W. While not significant, we observed a trend toward a reduction of the mEPSC in *Nacc1^+/−^* neurons expressing Nacc1-R284W. However, immunolabeling of autaptic neurons showed no evidence of a reduction in the number of synapses formed by neurons expressing this mutant. As such, the inhibition of glutamatergic synaptic transmission observed in neurons expressing Nacc1-R284W cannot be explained by a loss of synapses.

In addition to the changes in eEPSC amplitude and cell-surface glutamate receptor expression, presynaptic function was also perturbed by Nacc1-R284W expression when WT neurons (i.e., *Nacc1^+/+^*) were examined instead of *Nacc1^+/−^* neurons. Differences between the effects observed in WT and *Nacc1^+/−^* neurons expressing Nacc1-R284W indicate that both the total Nacc1 levels and the ratio between WT and mutant Nacc1 are important in synapse function in glutamatergic neurons, indicating a complex relationship between Nacc1-WT and Nacc1-R284W in neurons.

Differences in neuronal function, such as the reduction in the current evoked by applied glutamate, were generally only detected between neurons expressing exogenous Nacc1-WT and those expressing exogenous Nacc1-R284W, but not between neurons expressing Nacc1-R284W and neurons expressing EGFP alone. This is likely due to the tendency for exogenous expression of Nacc1-WT to mildly potentiate eEPSC amplitude, RRP size, and glutamate-induced and KA-induced currents (Supplementary Figure S5), whereas Nacc1-R284W negatively modulated these parameters (Figure 1; Supplementary Figure S5).

The mechanism by which Nacc1-R284W exerts these effects in neurons is not clear. The results presented here indicate that mutant Nacc1 can alter both pre- and postsynaptic function, depending on the relative levels of expression of WT and mutant Nacc1. Notably, ablation of Nacc1 expression does not impair glutamatergic transmission (Supplementary Figure S4), while expression of Nacc1-R284W significantly alters glutamatergic transmission when expressed in either WT or *Nacc1^+/−^* neurons (Figure 1; Supplementary Figure S5). Therefore, it is likely that Nacc1-R284W acts as a dominant negative rather than a loss of function mutation, with Nacc1-R284W acting antagonistically against the native function of Nacc1-WT. *In vivo* evidence also supports this hypothesis, in that Nacc1-KO mice lack gross neurological defects (Yap et al., 2013), yet humans that heterozygously express NACC1-R298W have profound abnormalities in brain structure and function. We therefore propose that Nacc1-R284W, and by extension NACC1-R298W, likely constitute dominant negative mutants.

### Nacc1 SUMOylation and its modulation by the R284W mutation

We performed a Y2H screen using a rat brain cDNA library and identified a range of novel Nacc1 interaction partners, including transcriptional regulators (Zmym2, ZBTB18, L3MBTL2, Casp8ap2, Atf7ip, Kat5, Zcch18), the SUMO-dependent E3 ubiquitin ligase Arkadia, the cytoplasmic proteins Gprasp1, Plk2, and SynGAP1, and four SUMO E3 ligases (PIAS1, PIAS2, PIAS3, and Ranbp2). In addition, protein sequence alignment revealed that all PIAS prey fragments identified in the screen contained the SP-RING domain (Supplementary Figure S6), which facilitates the transfer of the SUMO moiety to substrate proteins (Pichler et al., 2017). These data led us to further investigate the SUMOylation of Nacc1.

SUMOylation has attracted substantial interest in the field of neuroscience (Krumova and Weishaupt, 2012; Daniel et al., 2017; Bernstock et al., 2018; Stankova et al., 2018; Ripamonti et al., 2020; Ilic et al., 2022). While Nacc1 SUMOylation by SUMO2 had been detected in previous proteomics screens (Schou et al., 2014; Hendriks et al., 2015, 2018), our data validate *in vivo* the SUMOylation of Nacc1 in mouse brain and show that Nacc1 can be SUMOylated by both SUMO1 and SUMO2 *in vitro*. Strikingly, we also found that the SUMOylation of Nacc1-R284W is increased compared to Nacc1-WT in neurons. While the molecular mechanism remains to be determined, this phenomenon constitutes an unprecedented increase in the SUMOylation of a protein due to a disease-associated single amino acid mutation. The physiological impact of this increased Nacc1 SUMOylation is unclear, but it alters the Nacc1 interaction with SynGAP1 *in vitro*.

### The R284W mutation impairs Nacc1 interactions with GluK2A and SynGAP1

Co-immunoprecipitation analyses showed that, compared to Nacc1-WT, Nacc1-R284W exhibited greatly reduced binding to GluK2A and SynGAP1. The R284W mutation lies outside the known binding domains of Nacc1, which are the BTB domain, which mediates protein–protein interactions, and the BEN domain, which is thought to mediate both DNA and protein binding (Abhiman et al., 2008; Gao et al., 2020). These data indicate that the uncharacterized region of Nacc1 between the BTB and BEN domains undergoes a structural alteration due to the R284W mutation, which disrupts binding to GluK2A and SynGAP1. To predict the three-dimensional structure of Nacc1, we used AlphaFold-Multimer which yielded five different models each for human and mouse Nacc1 (Supplementary Figure S1C). Apart from the BEN and BTB domains, which have high confidence prediction (see predicted aligned error graph, Supplementary Figure S1C), all other regions have low prediction confidence. Therefore, it is currently not possible to accurately predict how the Nacc1-R284W/NACC1-R298W mutation might affect the structure, folding, and stability of the Nacc1 protein. Additionally, it is not possible to accurately predict how close the SUMOylation sites and the patient point-mutation would be to each other. However, the Nacc1-R284W/NACC1-R298W mutation is located in the middle of a predicted alpha-helix, and may thus cause a structural rearrangement. Such a structural change could then form the basis for altered SUMOylation and protein–protein interactions in mutant Nacc1.

While Nacc1-R284W exhibits increased SUMO conjugation, our data indicate that Nacc1 binding to GluK2A is independent of SUMOylation. In contrast, the increased SUMOylation of Nacc1-R284W may explain the reduced binding of Nacc1-R284W to SynGAP1. Moreover, the reduced binding of Nacc1-R284W to GluK2A and SynGAP1 may be associated with the defects in glutamatergic transmission observed in neurons expressing the mutant, particularly given the central role of SynGAP1 in regulating synaptic strength, glutamate receptor localization, and spine formation (Vazquez et al., 2004; Rumbaugh et al., 2006; Muhia et al., 2012; Araki et al., 2015; Llamosas et al., 2020). While so far there is little evidence for the presence of Nacc1 at synapses, NACC1/Nacc1 is present in extra-nuclear compartments of neurons, such as synaptic cytosol (Korutla et al., 2005, 2009; Shen et al., 2007), i.e., in compartments that also contain SynGAP1 and GluK2A (Supplementary Figure S2E; Ball et al., 2010; Qiu et al., 2018; Araki et al., 2020; Gou et al., 2020). Thus, it is possible that interactions between Nacc1 and SynGAP1 or GluK2A take place as the latter proteins are in transit to or from synapses.

Interestingly, while Nacc1 was not identified in a proteomics screen that identified the SynGAP1 interactome at the postsynaptic density (Wilkinson et al., 2017), we identified polo-like kinase 2 (Plk2) as a Nacc1 binding partner in our yeast two-hybrid screen (Table 1; Supplementary Figure S6). SynGAP1 is a substrate of Plk2 (Walkup et al., 2016, 2018), and Plk2 is implicated in homeostatic synaptic plasticity (Seeburg et al., 2008), a process that critically involves cell-surface AMPA receptor levels. Altogether, these data indicate that Nacc1 interacts with several key proteins involved in glutamatergic transmission. However, further work beyond the scope of the current study is required to comprehensively determine how the R284W mutation alters the structure of Nacc1, the Nacc1 interactome, and the function of Nacc1 in neurons.

### Implications of this study for disease

We report here the effects of the first disease-associated Nacc1 mutant on neurotransmission and neuronal function. SynGAP1 and GluK2A are two major components of the post-synaptic density and key players in synapse function and plasticity. Neurons expressing Nacc1-R284W on a *Nacc1^+/−^* genetic background exhibited reduced KA-induced current, likely indicating reduced KAR surface expression. KARs modulate neuronal circuit activity and are associated with experimental models of epilepsy (Contractor et al., 2011; Lerma and Marques, 2013; Crepel and Mulle, 2015). Thus, the prevalence of epilepsy in patients expressing NACC1-R298W raises the possibility that aberrant regulation of GluK2A may play a role in the patients’ symptoms. As regards SynGAP1, human mutations are known to cause the SynGAP syndrome, which is characterized by developmental delay and intellectual disability (Kilinc et al., 2018; Holder et al., 2019; Gamache et al., 2020), and mice lacking SynGAP1 exhibit mislocalization of AMPARs (Vazquez et al., 2004; Muhia et al., 2012). Thus, there is a possible functional overlap between SynGAP1 as a regulator of glutamate receptor trafficking and our present data, which indicates reduced cell surface expression of glutamate receptors in neurons expressing Nacc1-R284W.

## Limitations of the study

In autaptic neurons, Nacc1-R284W causes abnormal excitatory neurotransmission in a dominant-negative manner. At the biochemical level, Nacc1-R284W exhibits increased SUMOylation and reduced interaction with SynGAP1 and GluK2A, but the absence of Nacc1-R284W SUMOylation only reverts its interaction with SynGAP1 to WT levels. Our work revealed a complex relationship between Nacc1-WT, Nacc1-R284W, their respective SUMOylated forms, and SynGAP1 or GluK2A. Theses interactions were primarily assessed using proliferating non-neuronal cells, which may not necessarily reflect interactions in more complex cells, such as neurons *in vivo*, or specific subcellular compartments, such as synapses. Therefore, further analyses using intact brain tissue are required to fully determine how the R284W mutation in Nacc1 alters its biochemical behavior, its interacting proteome, or its cellular distribution and thus leads to perturbed synapse function.

## Conclusion

We report the first evidence that NACC1 plays a role in regulating glutamatergic neurotransmission, which is compromised in neurons expressing a disease-associated mutant form of the protein, Nacc1-R284W, likely by exerting a dominant negative effect. The R284W mutation leads to increased SUMOylation and loss of interaction with the key synaptic proteins GluK2A and SynGAP1. This work provides the first insights into the functional impact of a *de novo* mutation in NACC1 identified in patients with intellectual disability.

## Data availability statement

The original contributions presented in the study are included in the article/Supplementary material, further inquiries can be directed to the corresponding authors.

## Ethics statement

The animal study was reviewed and approved by Niedersächsisches Landesamt für Verbraucherschutz und Lebensmittelsicherheit (LAVES).

## Author contributions

MT, JD, and NB conceived the project, designed experiments, and wrote the manuscript with the help of all other authors. JD and SE performed experiments and analyzed data. JW provided the Nacc1-KO mouse model. JR provided protocols and resources. All authors contributed to the article and approved the submitted version.

## Funding

This work was supported by the German Research Foundation (SFB1286/A9, MT and NB). They would also like to acknowledge that their molecular graphics and analyses were performed with UCSF ChimeraX, developed by the Resource for Biocomputing, Visualization, and Informatics at the University of California, San Francisco, with support from National Institutes of Health R01-GM129325 and the Office of Cyber Infrastructure and Computational Biology, National Institute of Allergy and Infectious Diseases.

## Conflict of interest

The authors declare that the research was conducted in the absence of any commercial or financial relationships that could be construed as a potential conflict of interest.

## Publisher’s note

All claims expressed in this article are solely those of the authors and do not necessarily represent those of their affiliated organizations, or those of the publisher, the editors and the reviewers. Any product that may be evaluated in this article, or claim that may be made by its manufacturer, is not guaranteed or endorsed by the publisher.

## Supplementary material

SUPPLEMENTARY FIGURE S1 Alignment of human and mouse Nacc1 sequences. **(A)** Sequence alignment of human and mouse Nacc1. The human R298W mutant is the disease-associated variant identified in human patients. Sequence alignment shows that introducing an R to W mutation at amino acid position 284 in mouse Nacc1 results in a mutation equivalent to the *de novo* human mutation (red triangle). The range of amino acids covered by each sequence fragment is shown adjacent to the sequence name. **(B)** Schematic of the human (top numbers) and murine (bottom numbers) Nacc1 mutants used in this study. Nacc1 possesses two known domains and two lysines that are subject to SUMO-conjugation. Nacc1-R284W bears a disease-associated single amino acid mutation. In Nacc1-2KR both lysines K167 and K485 have been mutated to arginines, removing the sites of SUMO conjugation. Schematics are to scale with respect to the full length of Nacc1. **(C)** Cartoon representation of AlphaFold-based models for the tertiary structures of human (left) and mouse (right) Nacc1. For each species, five structure models were generated with AlphaFold-Multimer (Jumper et al., 2021). The location of the BTB and BEN domains are highlighted in blue and green, respectively. The N- and C-terminal residues are indicated. The R284W mutation in mouse and R298W mutation in human are indicated in red. The two SUMO acceptor lysines are indicated in orange. The boxes indicate the predicted aligned error scores of the respective Nacc1 model. The structures were visualised with ChimeraX (Goddard et al., 2018; Pettersen et al., 2021).

SUPPLEMENTARY FIGURE S2 Nacc1 expression level in neurons cultured from *Nacc1*^+/−^ mice. **(A)** Total protein stain (bottom panel) and anti-Nacc1 Western blot analysis (top panel) of cultured hippocampal neuron lysates (14 DIV) from littermate Nacc1^+/+^ and Nacc1^+/−^ mice. A black bracket indicates the position of Nacc1-positive bands. **(B)** For quantification, the amount of Nacc1 in each lane was normalized to the total protein content of the corresponding lane (bottom panel in **A**) and then expressed as a proportion of the amount of Nacc1 in samples from Nacc1^+/+^ mice. Cell lysate from Nacc1^+/−^ neurons contained 52.3 ± 0.07% of the Nacc1 present in lysates of Nacc1^+/+^ neurons. Data were compared using an unpaired Welch *t*-test. ** denotes a p value of between 0.001 and 0.01. Error bars represent SEM, N = 3 x Nacc1^+/+^, 4 x Nacc1^+/−^ mice. Molecular weights in kDa are indicated next to the blots. **(C)** Bar charts showing the cell membrane capacitance in individual neurons transduced as indicated below the graph. In each chart, dots represent values for individual cells, while the bar height represents the median of all cells. Data were compared using a Kruskal-Wallis test by ranks followed by Dunn’s multiple comparisons test. **, *p* = 0.001–0.01. **(D)** Bar charts showing the average NMDA current normalized to eEPSC amplitude in individual neurons transduced as indicated below the graph. In each chart, dots represent values for individual cells, while the bar height represents the median of all cells. The number of cells analysed for each condition is displayed beneath the condition label on each chart. **(E)** Anti-Nacc1, SynGAP1 and GluK2A Western blot analysis of subcellular fractions from WT mouse brain. Subcellular fractions are designated as follows: *H*, whole brain homogenate; P1, nuclear pellet; P2, crude synaptosomal pellet; S2, crude synaptic cytosol. Western blotting for PSD95 and Synaptobrevin2 validates the fractionation. Black arrow indicates the position of Nacc1. Molecular weights in kDa are indicated next to the blots.

SUPPLEMENTARY FIGURE S3 Expression of Nacc1-WT or Nacc1-R284W has no impact on the number of synapses formed per autaptic neuron. Autaptic glutamatergic hippocampal neurons were cultured from WT mice and transduced with lentivirus inducing expression of either EGFP, Nacc1-WT, or Nacc1-R284W. Synapses were then counted by immunolabelling fixed neurons against VGLUT1 (presynaptic) and Shank2 (postsynaptic) **(A)** Representative images from autaptic neurons infected with each virus, as indicated, and immunolabelled against VGLUT1, Shank2, and EGFP. Regions of interest are outlined as white boxes. **(B)** Regions of interest, outlined as white box in A, are shown in detail to highlight individual synapses, with VGLUT1 in green and Shank2 in red. Examples of co-localization are indicated (white arrowheads). **(C)** Puncta exhibiting VGLUT1- Shank2 co-localization (synapses) were counted for each neuron. No significant difference is observed between the three conditions. Data were compared using a Kruskal-Wallis test by ranks followed by Dunn’s multiple comparisons test. In each chart, dots represent values recorded for individual cells, while the bar height represents the median of all cells. The number of cells analysed for each condition is as follows: EGFP = 5 cells, Nacc1-WT = 5 cells, Nacc1- R284W = 7 cells.

SUPPLEMENTARY FIGURE S4 Nacc1-KO neurons exhibit reduced vesicular release probability but no change in evoked glutamatergic transmission. Autaptic glutamatergic hippocampal neurons were cultured from WT or Nacc1-KO mice and synaptic function was examined using patch clamp recording. Bar charts showing the measurement of eEPSC amplitude **(A)**, sucrose-evoked RRP charge **(B)**, eEPSC charge **(C)**, Pvr **(D)**, mEPSC amplitude **(E)**, mEPSC frequency **(F)** and glutamate-mediated current **(G)** in autaptic, glutamatergic hippocampal neurons from Nacc1-KO mice, compared to neurons from WT littermates. Bar height represents the median of all cells. The number of cells analysed for each condition is displayed beneath the condition label on the charts. Data were compared using a Mann-Whitney U test. *Denotes a *p* value of between 0.01 and 0.05.

SUPPLEMENTARY FIGURE S5 Expression of Nacc1-R284W impairs excitatory synaptic transmission compared to Nacc1-WT in Nacc1^+/+^ glutamatergic neurons. Autaptic glutamatergic hippocampal neurons were cultured from WT mice and transduced with lentivirus inducing expression of either EGFP (black), Nacc1-WT (blue), or Nacc1-R284W (red). Synaptic function was then examined in transduced neurons using patch clamp recording. **(A)** Overlaid representative eEPSC traces taken from a single cell from each transduction condition. **(B)** Bar charts showing the eEPSC amplitudes measured from single autaptic neurons transduced as above. **(C)** Overlaid representative traces of the current induced by perfusion with 100 μM glutamate. **(D)** Bar charts showing the amplitude of the peak current generated by glutamate application in transduced neurons. **(E)** Overlaid representative traces of the current induced by perfusion with 10 μM kainic acid (KA). **(F)** Bar charts showing the amplitude of the peak current generated by KA application in transduced neurons. **(G)** Bar charts showing the amplitude of spontaneous miniature EPSCs (mEPSCs) recorded from individual transduced neurons in the presence of 300 nM TTX. **(H)** Bar charts showing the frequency (Hz) of mEPSCs recorded from individual transduced neurons. **(I)** Representative overlaid traces of the current evoked by the application of 500 mM sucrose to infected neurons to evoke fusion of SVs in the RRP. **(J)** Bar charts showing the total charge transferred by the release of the RRP in individual neurons transduced as above. **(K)** Bar charts showing the charge transferred by the eEPSC in individual neurons. **(L)** Bar charts showing the probability of vesicular release (Pvr) individual neurons transduced as above. Pvr was calculated by dividing the charge transfer during an evoked EPSC by the charge transfer during the sucrose response, and then expressed as a percentage. In each chart, dots represent values recorded for individual cells, while the bar height represents the median of all cells. The number of cells analysed for each condition is displayed beneath the condition label on each chart. Data were compared using a Kruskal-Wallis test by ranks followed by Dunn’s multiple comparisons test. *Denotes a *p* value of between 0.01 and 0.05, ** denotes a *p* value of between 0.001 and 0.01, *** denotes a *p* value of between 0.0001 and 0.001.

SUPPLEMENTARY FIGURE S6 Schematic showing validated prey fragments and full-length prey proteins. Schematic showing the primary structure of validated prey fragments from Table 1 (red) above the corresponding full-length protein (black). Below the full-length protein are indicated the positions of known protein-protein interaction domains (green), SUMO-interaction motifs (thick blue bars) and the positions of SUMOylated lysines (light blue circles). Validated prey proteins were divided into categories based on known functions or sub-cellular localization, as indicated to the right of the sequences. Protein fragments are not to scale and the length of each rat protein sequence is indicated. Alignment was performed using sequences from the Universal Protein Resource.

SUPPLEMENTARY FIGURE S7 Nacc1 binds to PIAS2. **(A)** Anti-Nacc1 (top panel) and anti-HA (bottom panel) Western blot analysis of input and eluate fractions from an anti-HA affinity purification (IP: anti-HA) in N2A cells that had been transfected with HA-PIAS2 alone or in combination with Nacc1-WT. Endogenous Nacc1 was detected in the input samples (first lane) and transfection with Nacc1-WT produced mild overexpression of the protein (top panel) in the experiment depicted here. A black arrowhead indicates unmodified Nacc1 (top panel) or HA-PIAS2 (bottom panel), and a white arrowhead indicates SUMOylated Nacc1. **(B)** Anti-HA (top panel) and anti-Nacc1 (bottom panel) Western blot analysis of input and eluate fractions from an anti-myc affinity purification (IP: anti-myc) in N2A cells that had been transfected with HA-PIAS2 plus either various myc-Nacc1 constructs or empty pcDNA3 vector, as indicated above the representative blots. A black arrowhead indicates HA-PIAS2 (top panel) or myc-Nacc1 (bottom panel). Molecular weights in kDa are indicated next to the blots. Blots are representative of 3 independent experiments.

SUPPLEMENTARY FIGURE S8 Full Western blot membranes from Figure 2. Uncropped membranes from the experiments described in Figure 2, demonstrating the interaction between Nacc1 and GluK2A **(A,B)**, or Nacc1 and SynGAP1 **(C,D)**. Lanes are labelled according to the transfection conditions used for each sample. In **A** and **B**, cell lysates were subjected to anti-HA immunoprecipitation and then immunoblotted with anti-GluK2A/3 **(A)** and anti-HA **(B)**. In **C** and **D**, cell lysates were subjected to anti-SynGAP1 immunoprecipitation and then immunoblotted with anti-HA **(C)** and anti-SynGAP1 **(D)**. Molecular weights in kDa are indicated next to the blots.

SUPPLEMENTARY FIGURE S9 Anti-HA immunoblotting of membranes from Figure 3. Anti-HA immunoblotting of the membranes shown in Figures 3B,C. **(A)** Anti-HA Western blot analysis of input and eluate fractions from an anti-HA affinity purification (IP: anti-HA) in N2A cells expressing HA-SUMO1 plus either EGFP, Nacc1-WT, Nacc1-K167R, Nacc1- K485R, or Nacc1-2KR. Enrichment of HA-SUMO1 was evident in the immunoprecipitation samples. **(B)** Anti-HA Western blot analysis of input and affinity purification eluate fractions (IP: anti-HA) of N2A cells expressing HA-SUMO2 plus either EGFP, Nacc1-WT, Nacc1- K167R, Nacc1-K485R, or Nacc1-2KR. Molecular weights in kDa are indicated next to the blots.
