# Supplementary material for: An intellectual-disability-associated mutation of the transcriptional regulator NACC1 impairs glutamatergic neurotransmission
Source: Front Mol Neurosci. 2023 Jul 14;16:1115880. doi: 10.3389/fnmol.2023.1115880 (PMC10393139; doi:10.3389/fnmol.2023.1115880)

Supplementary Figure 1

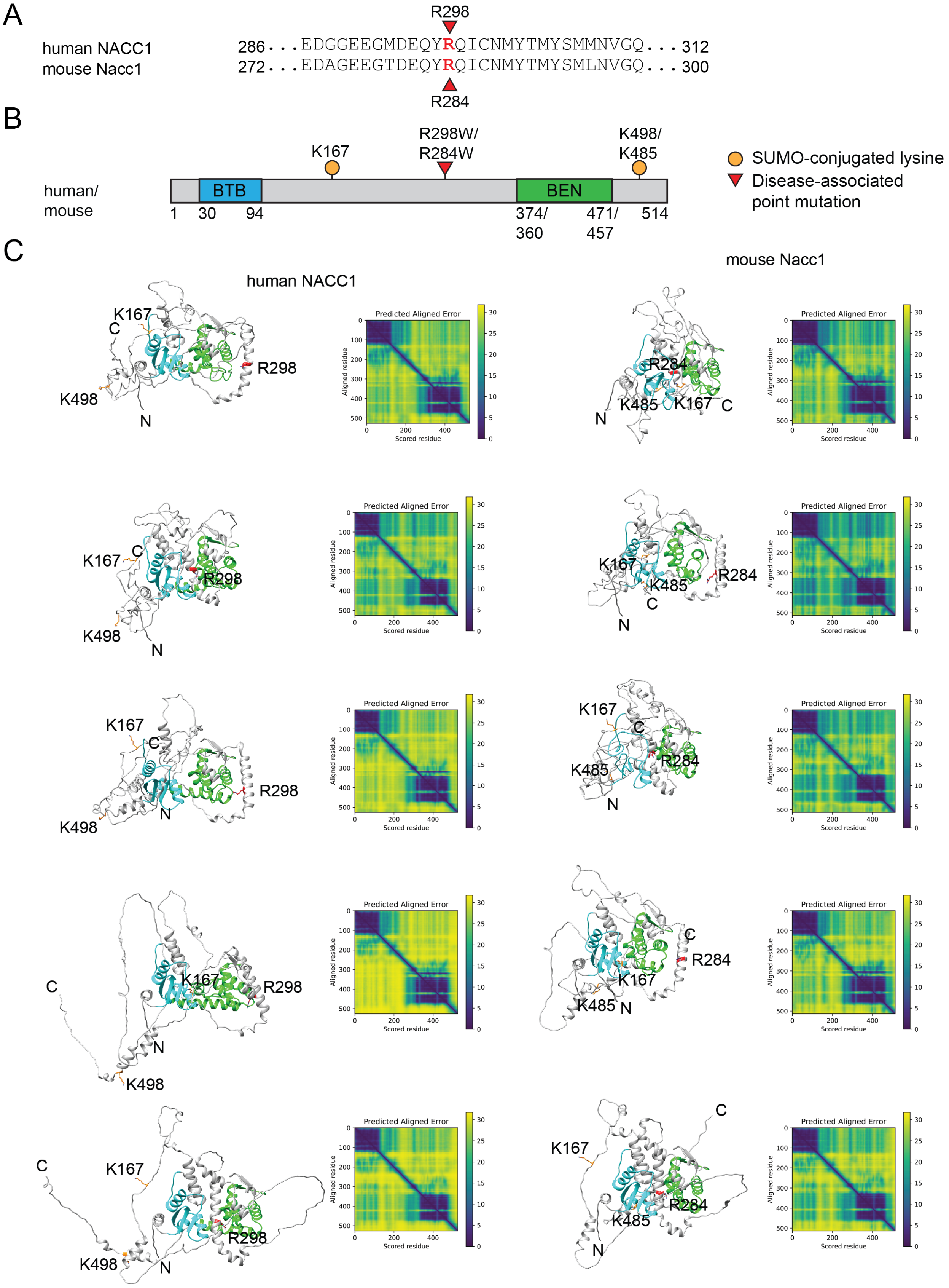

Supplementary Figure 2

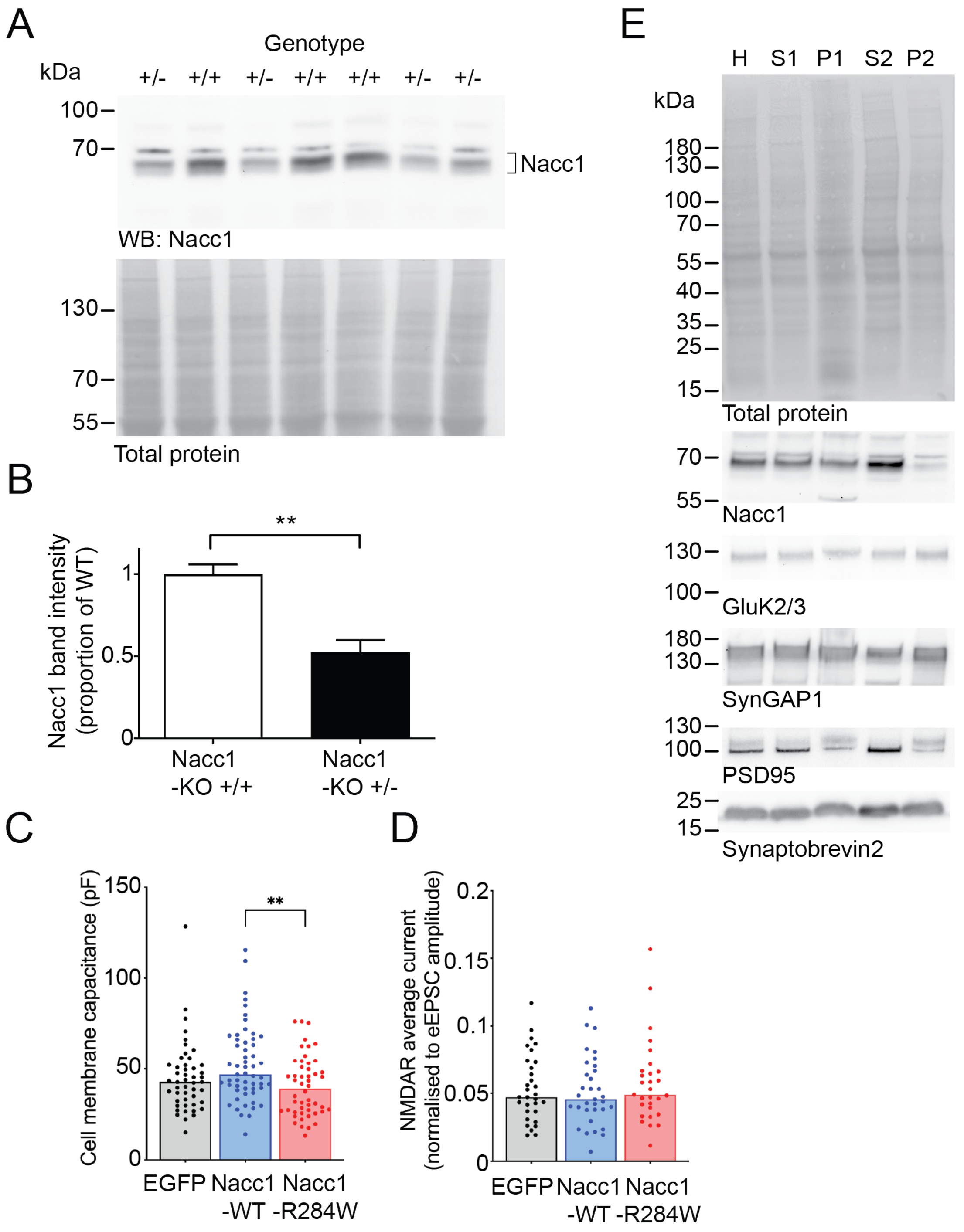

Supplementary Figure 3

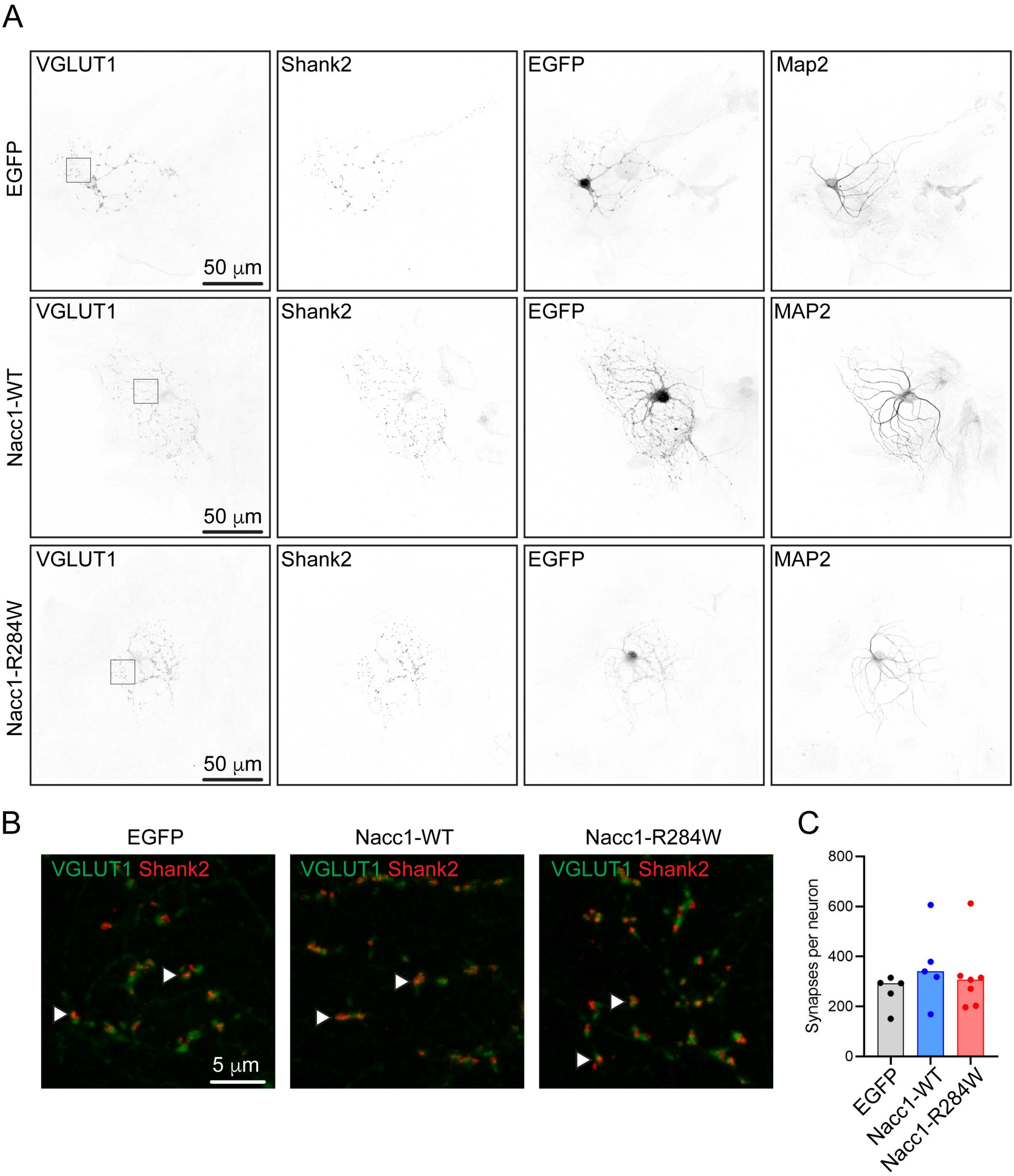

Supplementary Figure 4

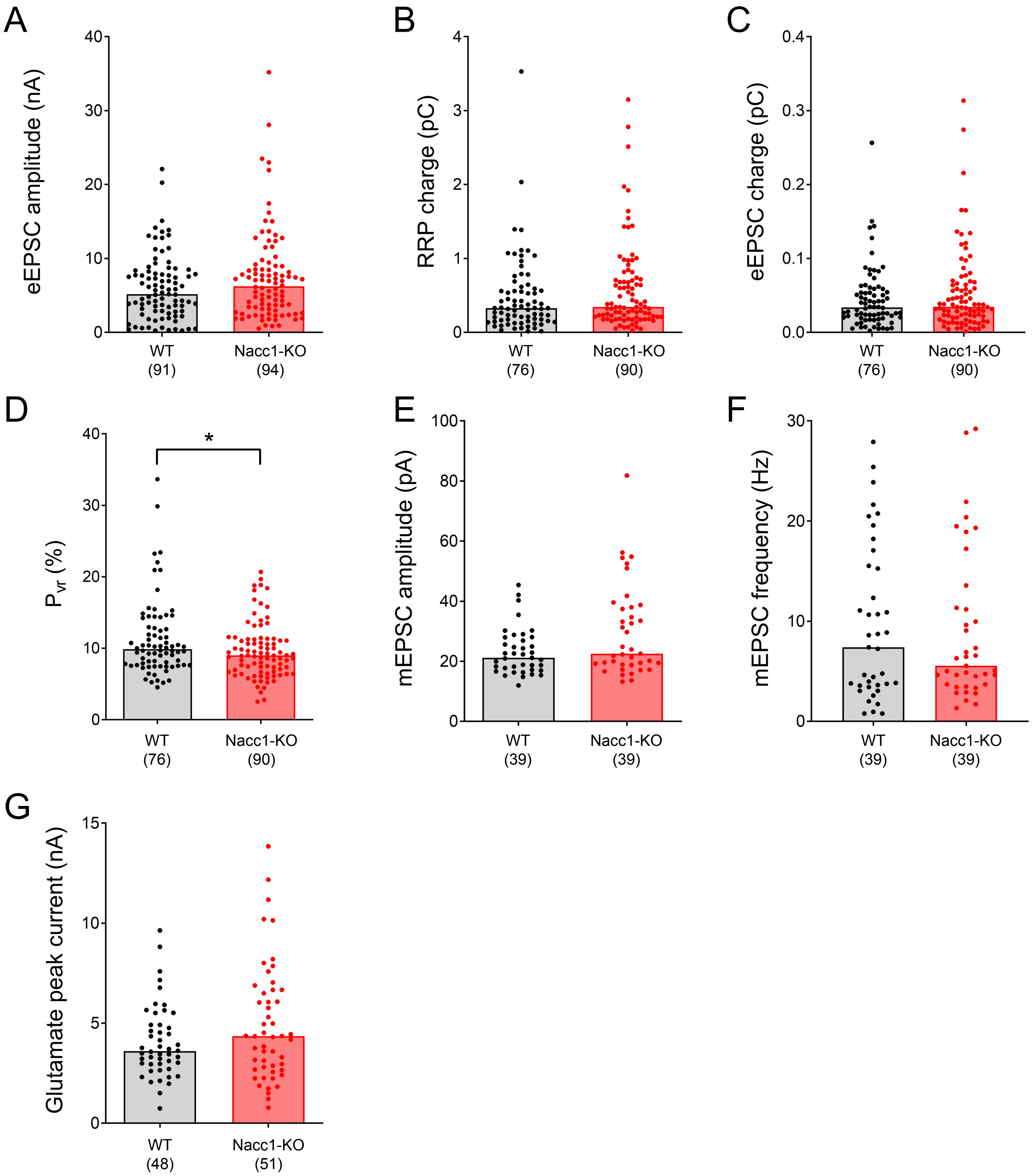

Supplementary Figure 5

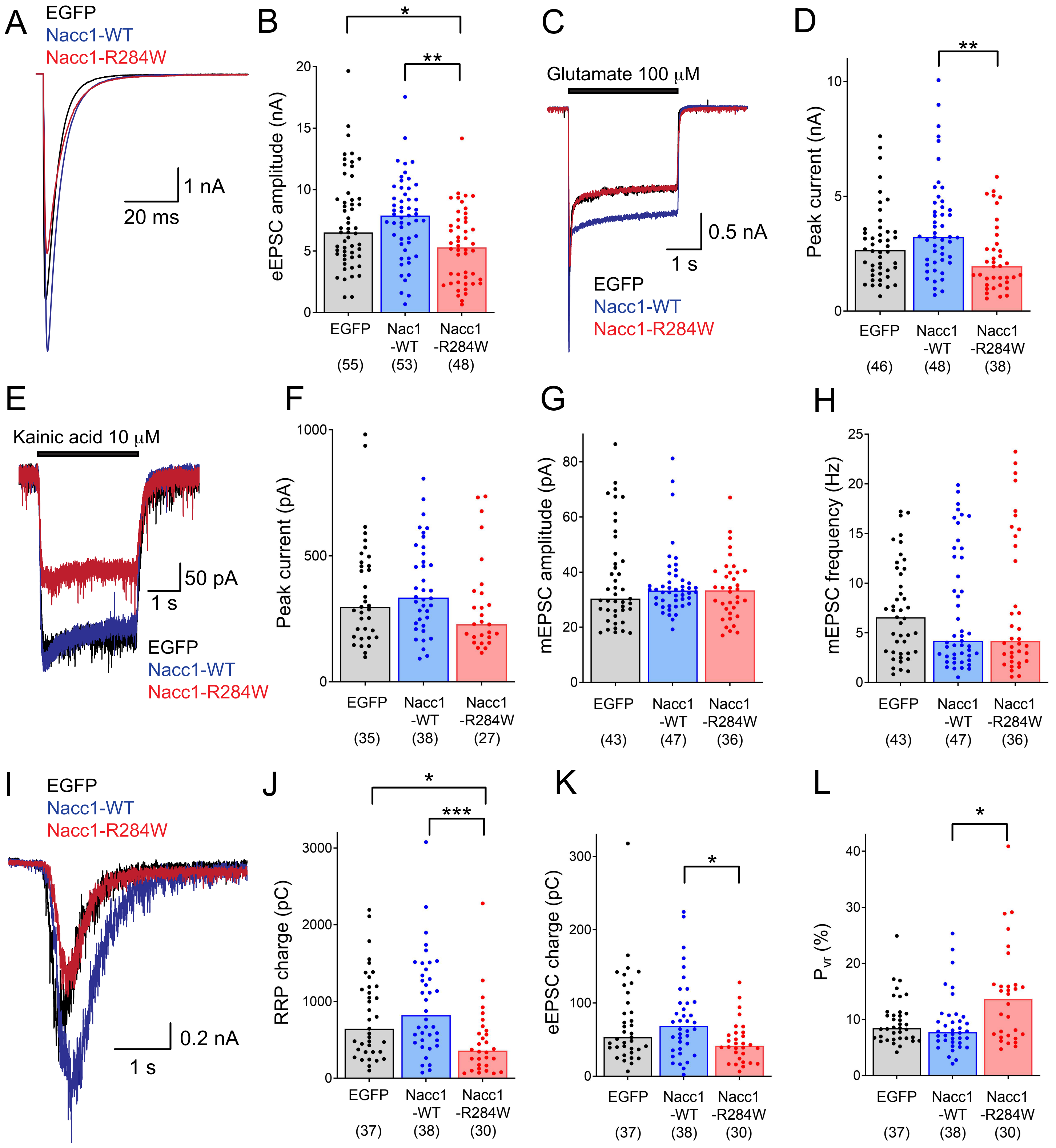

Supplementary Figure 6

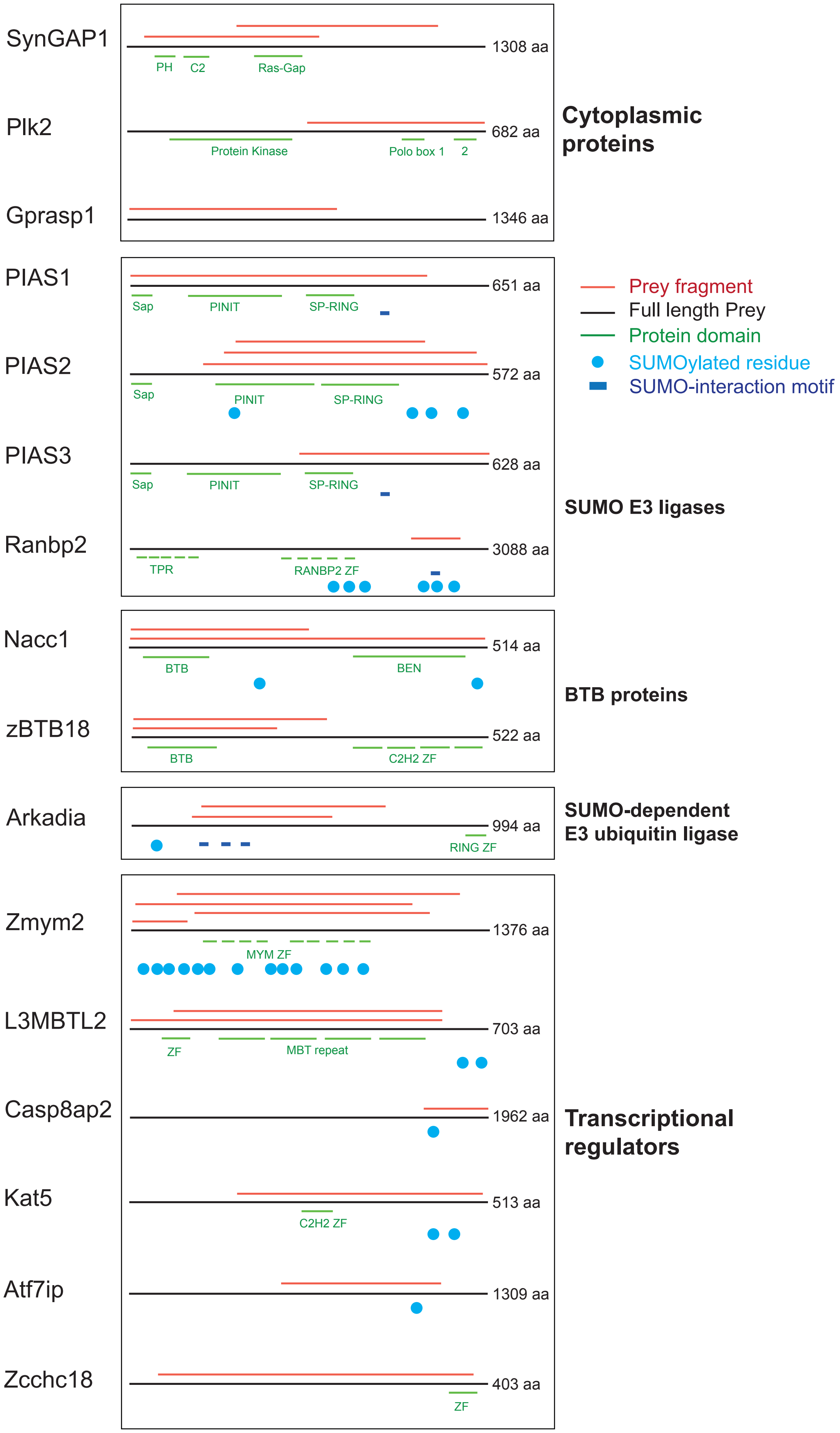

Supplementary Figure 7

A

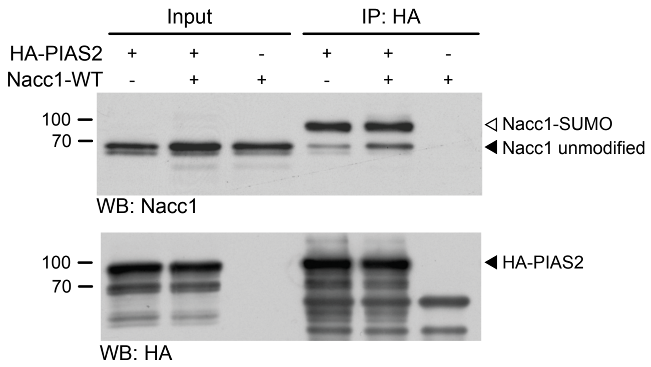

B

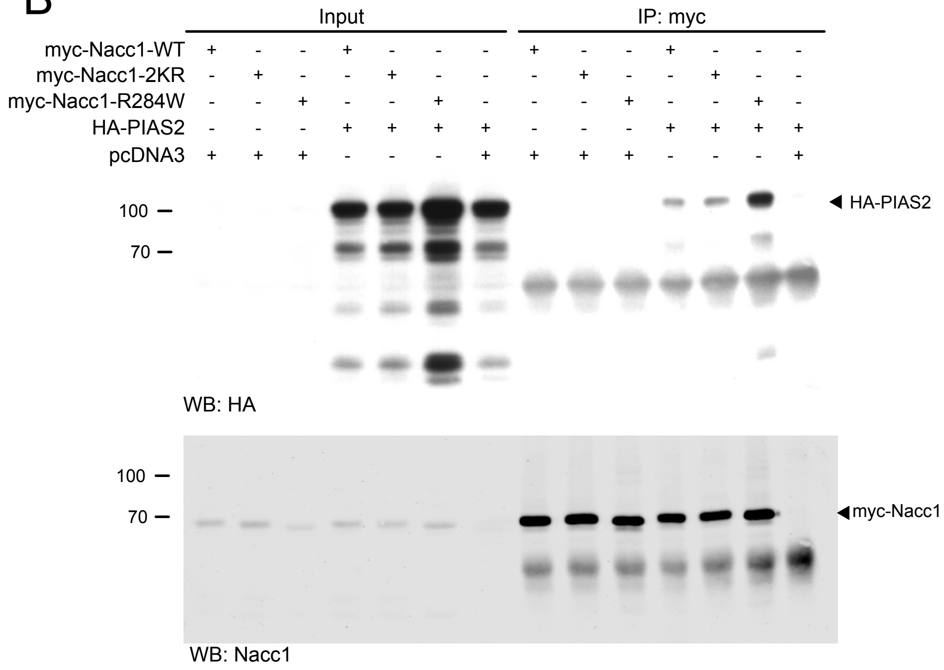

Supplementary Figure 8

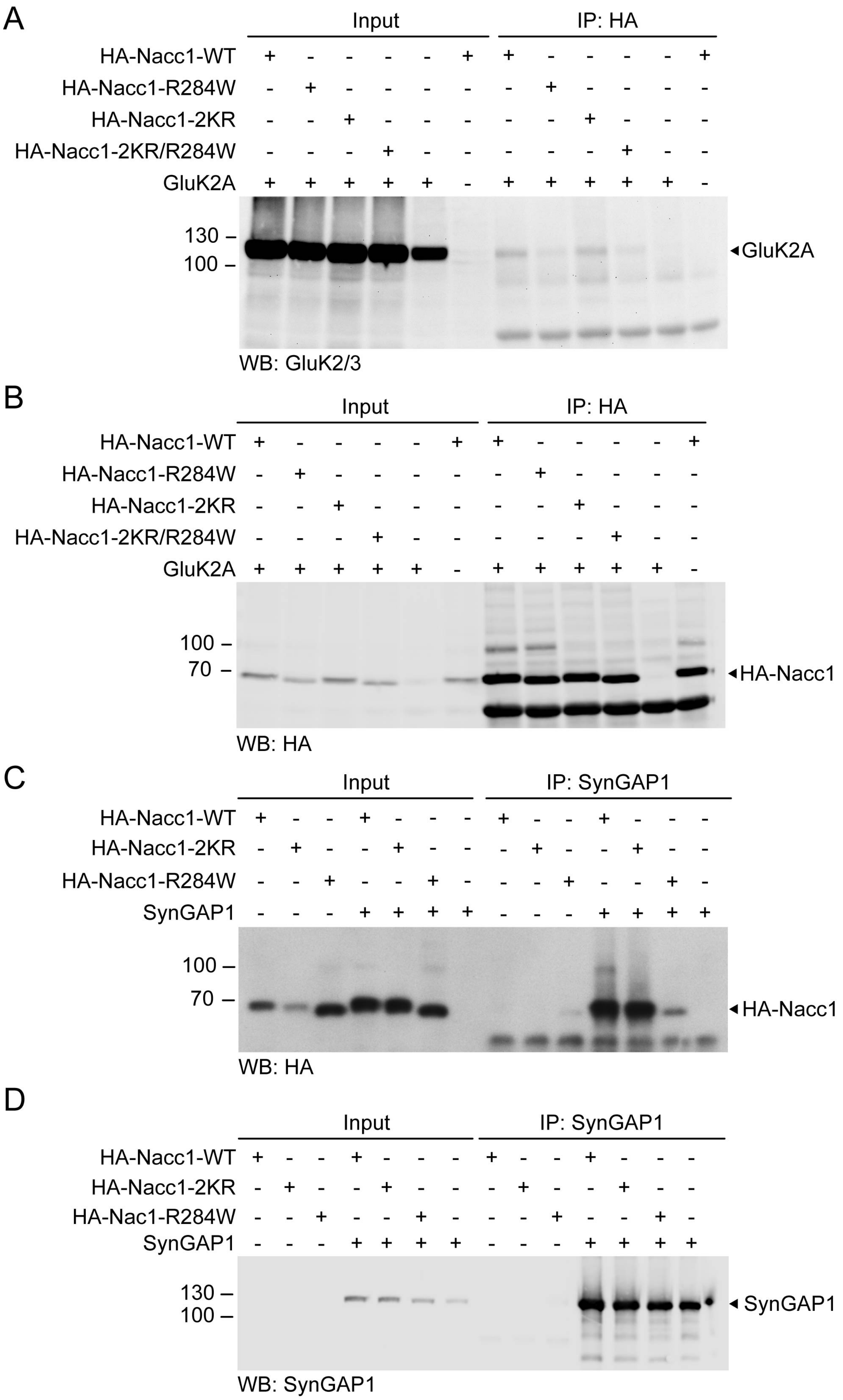

Supplementary Figure 9

A

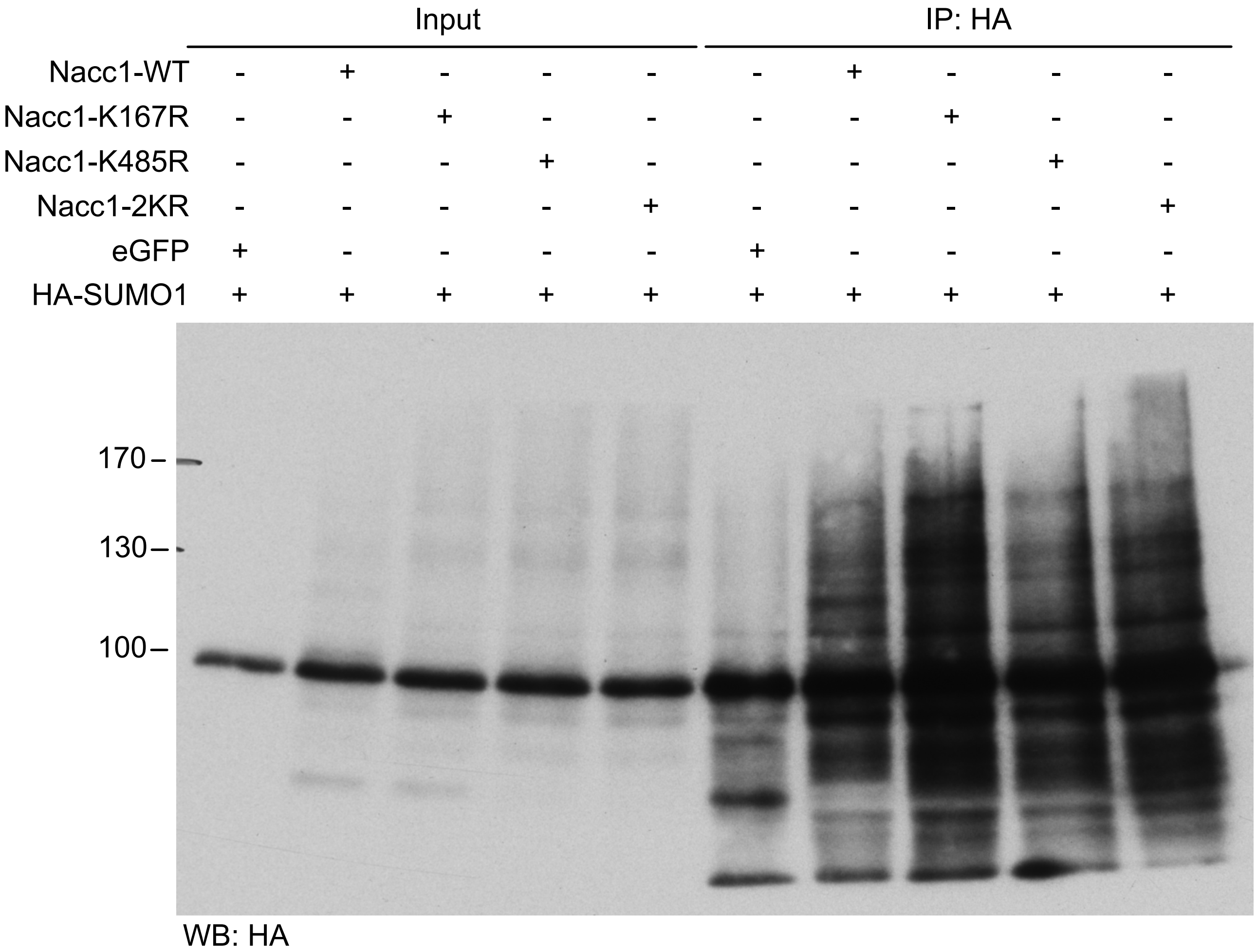

B

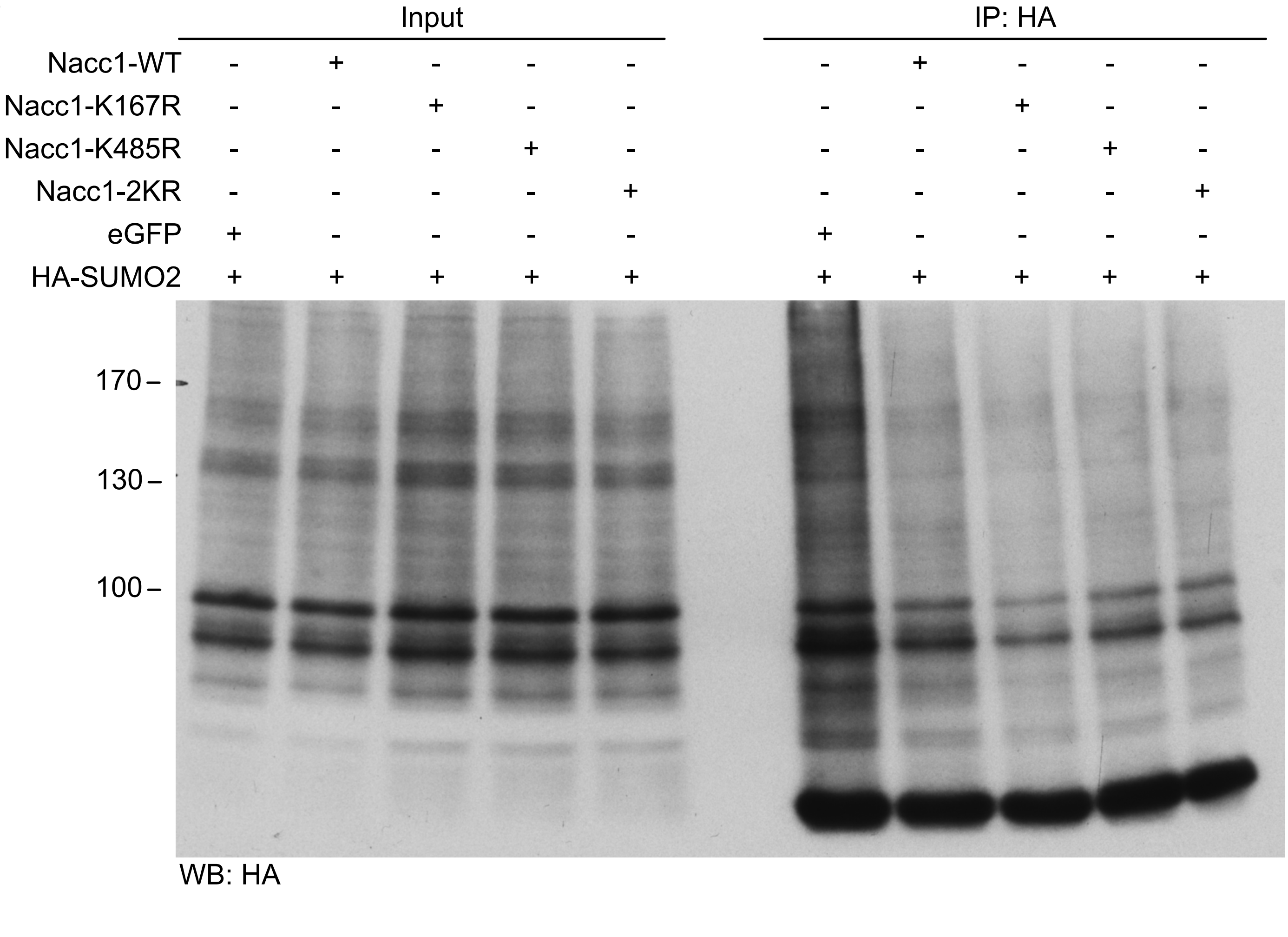

Supplement: SUPPLEMENTARY FIGURE S1 — Alignment of human and mouse Nacc1 sequences. (A) Sequence alignment of human and mouse Nacc1. The human R298W mutant is the disease-associated variant identified in human patients. Sequence alignment shows that introducing an R to W mutation at amino acid position 284 in mouse Nacc1 results in a mutation equivalent to the de novo human mutation (red triangle). The range of amino acids covered by each sequence fragment is shown adjacent to the sequence name. (B) Schematic of the human (top numbers) and murine (bottom numbers) Nacc1 mutants used in this study. Nacc1 possesses two known domains and two lysines that are subject to SUMO-conjugation. Nacc1-R284W bears a disease-associated single amino acid mutation. In Nacc1-2KR both lysines K167 and K485 have been mutated to arginines, removing the sites of SUMO conjugation. Schematics are to scale with respect to the full length of Nacc1. (C) Cartoon representation of AlphaFold-based models for the tertiary structures of human (left) and mouse (right) Nacc1. For each species, five structure models were generated with AlphaFold-Multimer (Jumper et al., 2021). The location of the BTB and BEN domains are highlighted in blue and green, respectively. The N- and C-terminal residues are indicated. The R284W mutation in mouse and R298W mutation in human are indicated in red. The two SUMO acceptor lysines are indicated in orange. The boxes indicate the predicted aligned error scores of the respective Nacc1 model. The structures were visualised with ChimeraX (Goddard et al., 2018; Pettersen et al., 2021). [file Data_Sheet_1.pdf]
